# Comparative Studies on Multi-Component Pharmacokinetics of *Polygonum multiflorum* Thunb Extract After Oral Administration in Different Rat Models

**DOI:** 10.3389/fphar.2021.655332

**Published:** 2021-06-17

**Authors:** Ninghui Ma, Yong Zhang, Liyan Sun, Yuan Zhao, Yue Ding, Tong Zhang

**Affiliations:** ^1^ School of Pharmacy, Shanghai University of Traditional Chinese Medicine, Shanghai, China; ^2^ Experiment Center of Teaching and Learning, Shanghai University of Traditional Chinese Medicine, Shanghai, China; ^3^ Experiment Center for Science and Technology, Shanghai University of Traditional Chines Medicine, Shanghai, China

**Keywords:** ANIT, CCl4, pharmacokinetics, bilirubin metabolism enzyme, metabolic transporter, polygonum multiflorum thunb

## Abstract

The clinical use of *Polygonum multiflorum* Thunb (PM) has been restricted or banned in many countries, due to its hepatotoxic adverse effects. Its toxicity research has become a hot topic. So far, the pharmacokinetic studies of PM, focusing on prototype compounds such as 2,3,5,4'-tetrahydroxystilbene-2-O-β-D-glucoside (TSG), emodin, and physcion, have been considered the main basis of pharmacodynamic material or of toxic effect. However, pharmacokinetic studies of its phase II metabolites have not yet been reported, mainly because the quantifications of such metabolites are difficult to do without the reference substance. In addition, pharmacokinetic studies on different pathological models treated with PM have also not been reported. On the other hand, toxic effects of PM have been reported in patients diagnosed with different liver pathologies. In the present work, a simultaneous quantitation method for eight prototypes components of PM and their five phase II metabolites has been performed by ultra-high performance liquid chromatography-tandem mass spectrometry (UPLC-MS/MS) and used for the pharmacokinetic study of PM in two different liver pathological models in rats (normal, alpha-naphthylisothiocyanate (ANIT), and carbon tetrachloride (CCl_4_)). The results showed that the main blood-entering components of PM are TSG, emodin, physcion, emodin-8-O-β⁃D⁃glucoside (E-Glu), physcion-8-O-β⁃D⁃glucoside (P-Glu), aloe-emodin, gallic acid, resveratrol and catechin, among which TSG, emodin, and catechin were primary metabolized in phase II, while resveratrol was converted to all phase II metabolites, and the others were metabolized as drug prototypes. Meanwhile, their pharmacokinetic parameters in the different models also exhibited significant differences. For instance, the AUC (0-∞) values of the TSG prototype and its phase II metabolites were higher in the ANIT group, followed by CCl_4_ group and the normal group, while the AUC (0-∞) values of the emodin prototype and its phase II metabolites were higher in the CCl_4_ group. To further illustrate the reasons for the pharmacokinetic differences, bilirubin metabolizing enzymes and transporters in the liver were measured, and the correlations with the AUC of the main compounds were analyzed. TSG and aloe-emodin have significant negative correlations with UGT1A1, BSEP, OATP1A4, OCT1, NTCP, MRP2 and MDR1 (*p* < 0.01). These data suggest that when the expression of metabolic enzymes and transporters in the liver is inhibited, the exposure levels of some components of PM might be promoted *in vivo*.

## Introduction

*Polygonum multiflorum* Thunb (PM) is a traditional tonic Chinese medicine used to fortify the liver and kidneys, benefit the essence and blood, and darken the hair. Also, it has been widely used in clinical and health care products (Medicine SAoTC, 1999; Commission CP, 2020). However, in recent years, there were increasing reports of adverse effects to the liver caused by PM, mainly manifested as hyperbilirubinemia, with classical clinical signs, such as yellow staining of the skin and sclera, and deepening of the urine color. Warning information on the hepatic injury promoted by PM has been released by Canadian, British and Australian as well as Chinese pharmacovigilance authorities (Zhang et al., 2009; LI C, 2015). We have reviewed the clinical literature reports on hepatic injury or hepatotoxic adverse reactions of PM in the past decade, and found that there are about 70 articles worldwide containing about 800 cases of adverse reactions of PM (Ma et al., 2020). The confusing use of crude and processed PM and large doses over the long term are the main factors causing adverse reactions in the liver caused by PM. Besides, some idiosyncratic susceptible populations should also receive more attention. There are two main mechanisms associated with the liver injury induced by PM. Firstly, genetic factors may predispose to the aggravation of adverse effects resulting from exposure to PM, for example carrying the HLA-B *35:01 allele (Li et al., 2019) and weak CYP450 activity in the body (Li et al., 2017). And secondly, PM may interfere with the expression of key proteins in the regulation of liver functions, such as the bilirubin metabolizing enzyme UGT1A1 expression inhibition (Qi et al., 2015; Wang et al., 2017; Qi et al., 2019), and the peroxisome proliferator-activated receptor-γ (PPAR-γ) inhibition (Lan-zhi et al., 2017).

Pharmacokinetic studies are an essential part of exploring the mechanisms related to liver injury caused by PM, taking into account the principal components (e.g., TSG and emodin) (Lv et al., 2011a; Lin et al., 2015a; Ma et al., 2015). The metabolites of these components in PM have been identified in some studies (Lin et al., 2015b; Ma et al., 2015; Zhang et al., 2018a; Zhang et al., 2018b). It is worth to mention that TSG, emodin, and physcion are mainly metabolized in phase II (glucuronidation and sulfation). However, the pharmacokinetic parameters of these metabolites have not been studied in the above reports, as the reference substances were hard to obtain. On that basis, we selected β-glucuronidase and sulfate esterase to hydrolyze the glucuronidated and sulfated combinations of plasma samples into the prototype drug. Then we measured the prototype drug indirectly to determine the phase II metabolites concentrations. It could help us to complete the gaps of pharmacokinetic studies for these phase II metabolites. Besides, liver injury caused by PM is often accompanied by an increase in the blood bilirubin levels, and the phase II metabolism of the above components is similar to the metabolism of bilirubin glucuronidation. It is hypothesized that the bilirubin metabolizing enzymes and transporters (e.g., UGT1A1, MRP2, BSEP, MDR1, and NTCP), which play important roles in the metabolism of bilirubin (Ransil et al., 1992; Faber et al., 2003; Otsuka et al., 2005; Hagenbuch, 2007; Nies et al., 2008; Hoekstra et al., 2013), would affect the pharmacokinetic behavior of the main components in PM. The liver injury profile induced by PM could be determined by the hepatocellular injury (*R* ≥ 5) and cholestatic/mixed liver injury (*R* < 5) according to the Roussel Uclaf Causality Assessment Method (RUCAM), with a high proportion of hepatocellular injury in clinical cases (Jung et al., 2011; Dong et al., 2014; Byeon et al., 2019; Liu et al., 2019; Wang et al., 2019). Bile duct ligation and drug induction are the most commonly used methods to simulate cholestasis in laboratory studies, with ANIT being the most common. CCl_4_ is also experimentally used as a common inducer of liver injury, and its induced pathological changes are primarily manifested as hepatocyte degeneration and necrosis. It is also described that UGT1A1 enzyme activity is impaired in ANIT and CCl_4_ intoxicated rats, similar to PM-induced liver injury (Sasaki et al., 1990; Chang et al., 2005; Zhao et al., 2007; Yang et al., 2008). Herein, it is the first work to investigate the different pharmacokinetic behavior of the PM components in the ANIT and CCl_4_-induced liver injury models in rats and the correlation between the exposure characteristics of the active ingredients in PM and the expression levels of liver metabolizing enzymes and transporters, which have given us some clues to illustrate the toxicity of PM in different rat models with liver injury.

## Materials and Methods

### Chemicals and Reagents

*Polygonum multiflorum* Thunb (PM) supplied by Shanghai Kangqiao TCM, Co., Ltd (Shanghai, China). The standards 2,3,5,4'-tetrahydroxystilbene-2-O-β-D-glucoside (TSG), emodin, aloe-emodin, physcion, emodin-8-O-β⁃D⁃glucoside (E-Glu), physcion-8-O-β⁃D⁃glucoside (P-Glu), gallic acid, catechin, resveratrol, and puerarin (≥98% purity) were all procured from the National Institute for the Control of Pharmaceutical and Biological Products (Beijing, China). Sulfatase was obtained from Helix pomatia (Type H-1, sulfatase≥10,000 units/g solid, Sigma, United States). The peptides LTIIPQDPILFSGSLR, GVALPETIEEAENLGR, AAATEDATPAALEK, TFQFPGDIESSK, LLLSGFQEELR, STALQLIQR, NTTGALTTR, EENLGITK, SVQPELK, and TYPVPFQR, as well as stable isotope-labeled internal standards (≥98% purity), were synthesized by Bankpeptide Biological Technology, Co., Ltd (Hefei, China). The ProteoExtract native membrane protein extraction kit was purchased from Calbiochem (Temecula, CA, United States). The BCA protein assay kit and in-solution trypsin digestion kit were obtained from Pierce Biotechnology (Rockford, IL, United States). Formic acid was MS grade; ammonium bicarbonate (98% purity) and sodium deoxycholate (98% purity) were purchased from Sinopharm Chemical Reagent, Co., Ltd (Shanghai, China). Acetonitrile and methanol, all MS grade, were obtained from Merk (Darmstadt, Germany). Acetonitrile and methanol, all MS grade, were obtained from Merk (Darmstadt, Germany).

### Preparation and Quality Control of *Polygonum multiflorum* Thunb Extract

**FIGURE 1:**
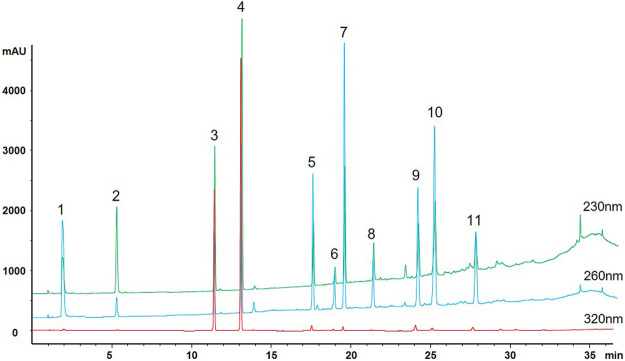
Multiple chromatograms of 11 constituents of PM (1. Gallic acid; 2. Catechin; 3. TSG; 4. Resveratrol; 5. Emodin-Glu; 6. Physcion; 7. Aloe-emodin; 8. Rhein; 9. Emodin; 10. Chrysophanol; 11. Physcion).

The alcoholic extract of *Polygonum multiflorum* Thunb (AE-PM) was prepared by immersing 90 g of PM in 540 ml of 75% ethanol (1:6, w/v) for 0.5 h. The sample was refluxed twice, each time for 1 h. The extraction solution was mixed and passed through a paper filter. Then, the filtrate was concentrated to 30 ml, and the final solution containing the crude drug presented a concentration of 3 g/ml. After that, we carried out a multicomponent assay, as described by Zhao MJ's (Zhao et al., 2017), on the PM, AE-PM, and aqueous extract of PM (QE-PM) for the following constituents: TSG, emodin, physcion, gallic acid, catechin, E-Glu, P-Glu, aloe-emodin, resveratrol. The results are shown in Figure 1 and Table 1. The chemical components of AE-PM were significantly more than that of the QE-PM, so we chose the AE-PM for our pharmacokinetic study.

**TABLE 1:** Content of active ingredients in PM and its different extracts. Data were expressed as mean ± SD.

Component	PM (mg/g)	QE-AM (mg/g)	AE-PM (mg/g)
TSG	102.62 ± 5.43	23.83 ± 2.54	72.80 ± 4.36^***^
Emodin	31.94 ± 2.66	6.04 ± 1.23	24.64 ± 1.52^***^
Physcion	8.42 ± 1.33	0.24 ± 0.01	8.53 ± 2.44^***^
Aloe-emodin	1.44 ± 0.56	\	0.90 ± 0.12
Catechin	2.36 ± 0.69	0.26 ± 0.04	2.85 ± 0.56^***^
E-glu	8.98 ± 1.01	0.19 ± 0.02	7.61 ± 1.05^***^
P-glu	1.24 ± 0.21	0.10 ± 0.01	1.40 ± 0.43^**^
Gallic acid	5.16 ± 1.55	0.32 ± 0.02	5.42 ± 1.48^***^
Resveratrol	7.15 ± 2.41	\	7.55 ± 1.63
Rhein	0.85 ± 0.12	\	0.66 ± 0.23
Chrysophanol	0.66 ± 0.23	\	0.50 ± 0.18

ANOVA test was used to calculate the significance of the differences, ***p < 0.001 and **p < 0.01 which compared with the QE-AM.

### Animals Handing

Male Sprague Dawley rats (200–220 g) were purchased from B&K laboratory Animal, Corp. Ltd (Shanghai, China), fed in the Laboratory Animal Center of Shanghai University of Traditional Chinese Medicine, and housed in an environmentally controlled animal room at a temperature of 22–24°C and a relative humidity of 60–65%. The animals were maintained on a 12:12 h light–dark cycle (lights on at 7:00 am) with regulated temperature and humidity. During the entirety of the experimental process, the rats were fed with certified standard rat chow and tap water *ad libitum*. All efforts were made to reduce animal suffering. The animal experiments strictly complied with the Guide for the Care and Use of Laboratory Animals, and the animal experiment protocols were approved by the Institutional Animal Committee of Shanghai University of Traditional Chinese Medicine (Permit No. PZSHUTCM19010406).

## Pharmacokinetic Studies of AE-PM in Different Rat Liver Disease Models

### Instrumentation and Chromatographic Conditions

**FIGURE 2:**
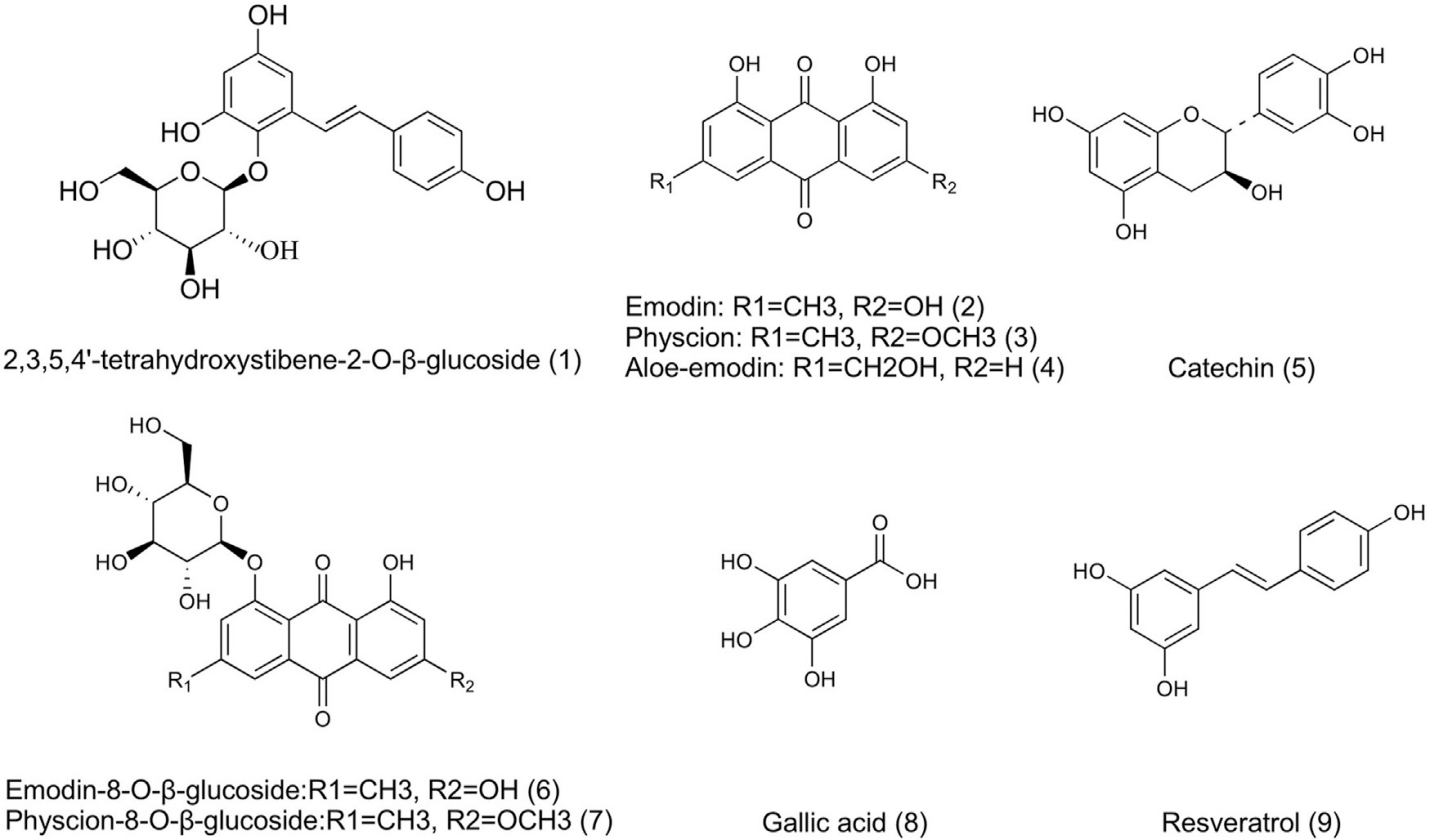
Chemical structures of 9 active components in AE-PM (1. TSG; 2. Emodin; 3. Physcion; 4. Aloe-emodin; 5. Catechin; 6. E-Glu; 7. P-Glu; 8. Gllic acid; 9. Resveratrol).

To explore the pharmacokinetic properties of PM following intragastric administration in rats, a rapid and sensitive method involving the use of UHPLC-MS/MS (Agilent 6460 series, Agilent Technologies, Santa Clara, CA, United States) was developed and validated for the simultaneous quantification of nine active components in rat plasma. The quantification was conducted in ESI negative ionization mode, and mass spectrometry conditions were set up as follows: capillary voltage of 3500 V; gas flow at 12 L/min; nebulizer of 40 psi; the gas temperature of 350°C; and delta EMV (−) of 400. A 10 µl extraction sample was injected into the column (Agilent SB-C18 column, 2.1 mm × 50 mm, 1.8 mm) and eluted at 0.4 ml/min with a gradient elution of water (with 0.1% v/v formic acid) (A) and acetonitrile (B) (0–1.5 min, 20–30% B; 1.5–3.5 min, 30–50% B; 3.five to four min, 50–55%B; 4–6.5 min, 55–85% B; 6.5–6.6 min, 85–20% B and re-equilibration for 3 min). Multiple reaction monitoring parameters and chemical structures of nine chemical components in PM (and internal standard) are shown in Table 2 and Figure 2.

**TABLE 2:** Multiple reaction monitoring parameters of nine chemical components in PM (and internal standard).

Component	Molecular weight	Parent ion	Product ion	Fragmentor	Collision energy (V)
TSG	406.39	404.9	243.1	156	27
Emodin	270.24	268.7	224.8	170	24
Physcion	284.27	283.1	240	170	23
Aloe-emodin	270.2369	268.9	239.6	150	24
Catechin	290.27	289	245.1	150	12
E-glu	432.11	430.9	268.8	165	27
P-glu	446.404	444.9	282.7	100	16
Gallic acid	170.12	168.9	125	110	11
Resveratrol	228.24	227	185	140	15
Puerarin (IS)	416.378	415	295	165	20

### Sample Preparation

A selective sample preparation method was applied to eliminate endogenous proteins’ interference and optimize extraction recovery. Among several chemical reagent methanol and acetonitrile, and acetonitrile combined with methanol and formic acid, were used. Moreover, the amounts of extraction solvent were all tested before. The application of triple methanol resulted in the highest sensitivity level and convenience, especially minimizing endogenous interference and enhancing extraction recovery. Therefore, we selected it as the optimal solvent for sample preparation.

The sulfatase chosen for this experiment contains β-glucuronidase activity, which could hydrolyze both glucuronide and sulfate conjugation metablites of components in PM into a prototype drug. According to the product information, the optimal working temperature of the sulfatase is 37°C, and the pH value if 5. According our previous results (Ding et al., 2012; Yi et al., 2018), the maximum hydrolysis of phase II metabolites was achieved when the sulfatase (330 units/ml) was incubated for 30 min at a volume ratio of 1:1 with the plasma sample.

A 50 µl aliquot of blood plasma sample was placed in a centrifuge tube with 50 µl of puerarin (internal standard, IS) solution (1,000 ng/ml), followed by 150 µl methanol, which was mixed for 5 min. Then, the mixture was centrifuged for 10 min at 15,000 rpm (4°C), the supernatant was transferred to a clean centrifuge tube and dried with nitrogen gas at room temperature. The residue was resuspended in 100 µl of methanol, then centrifuged at 15,000 rpm (4°C, 10 min), and 10 µl of the supernatant was analyzed by UHPLC-MS/MS.

Additional plasma samples (50 µl) were enzymatically hydrolyzed with 50 µl of enzyme solution (65.86 mg of sulfatase, dissolved in 2 ml of sodium acetate buffer, pH 5.0) for phase II metabolite quantification, in accordance with our previous study (Ding et al., 2012; Yi et al., 2018). After being vortex mixed for 5 min, the mixture was incubated at 37°C for 30 min and returned to room temperature. Subsequently, the samples were processed as described above.

## Method Validation

The UPLC-MS/MS method for determining the nine ingredients in rat blood plasma was validated according to the current US FDA Bioanalytical Method Validation Guidance (Guidance for Industry: Bioanalytical Method Validation, 2001) (Yuan et al., 2020). The following parameters were determined: specificity, linearity, lower limit of quantitation (LLOQ), accuracy, precision, extraction recovery, matrix effect, and stability.

### Specificity

The purpose of specificity analysis is to eliminate the interference of endogenous substances on the determination. Specificity was determined by comparing chromatograms of blank rat blood plasma obtained from six individual subjects with chromatograms of blood plasma samples obtained after AE-PM administration at a dose of 50 g/kg.

### Linearity

Calibration curves were constructed using the peak area ratios of the analytes to puerarin and by applying a weighted (1/x^2^) least squares linear regression analysis. The LLOQ was determined at the lowest concentrations at the signal-to-noize ratio (S/N) ≥ 10.

### Accuracy and Precision

Precision [expressed as the relative standard deviation (RSD)] and accuracy [expressed as the relative error (RE)] were calculated for three QC points (low, medium, and high). Six replicates of each QC point were analyzed to determine the interday accuracy and precision. This process was repeated three times over three consecutive days to determine the intraday accuracy and precision.

### Extraction Recovery and Matrix Effect

Recovery was evaluated in six replicates at three different QC concentrations (low, medium, and high). The percentage recovery was determined by comparing the concentrations of the pre-extraction spiked QC samples prepared in a blank matrix (by adding analytes and puerarin to blank matrix prior to extraction) with the peak area of the post-extraction spiked QC samples prepared in an extracted blank matrix (prepared by adding analytes and puerarin to blank matrix extract). Matrix effects were investigated on six independent sources of blank rat blood plasma by calculating the ratio of the peak area in the presence of matrix to the peak area in the absence of matrix at three different QC concentrations (low, medium, and high).

### Stability

The stability of standard analytes in rat plasma was evaluated under several conditions (time and temperature) by analyzing six replicates of the QC samples at three concentrations (low,medium, and high). Stability was investigated in terms of short and long-term stability, freeze and thaw stability, and post-preparative stability by using the developed method. Short-term stability was evaluated by storing QC samples at room temperature (25°C) for 24 h. Long-term stability was assessed after 60 days by a storage at −80°C. Freeze and thaw stability were determined after three freeze–thaw cycles at -80°C. In addition, post-preparative stability during storage in an auto sampler at 4°C for 24 h was investigated.

### Application to Pharmacokinetic Analysis

The rats were randomly distributed into three groups (*n* = 12 for each group: 1) normal group (group 1; 2) ANIT group (group 2; and 3) CCl_4_ group (group 3). Group 1 served as non-treated controls, whereas group 2 was treated with 4% ANIT at a dose of 50 mg/kg (diluted in olive oil) to induce cholestatic liver injury. Additionally, group 3 represented the CCl_4_-induced hepatocytes injury model, which was treated with pure CCl_4_ (5 ml/kg, s. c.) on day 0, 50% CCl_4_ (diluted in olive oil) (3 ml/kg, s. c.) on day 3, and 20% CCl_4_ (diluted in olive oil) (3 ml/kg, s. c.) on day 6. After 24 h, five rats selection for serum and liver collection from each of the three groups. Blood samples were collected in a coagulation tube, and were centrifuged at 4°C for 15 min (4,000 rpm), The resultant serum was used for the ALT, AST, TBIL, DBIL, TBA and ALP assays. Liver samples were dissected and stored at −80°C for further analysis, whereas the central part of the right large lobe of the liver was used for histological examination.

The remaining 21 rats of the three groups were treated with a single oral dose of AE-PM (50.4 g PM/kg). This dosage was chosen based on previous studies to liver injury in normal rats after long-term administration. Blood was collected in heparinized tubes at 5, 15, 30, 45, 60, 120, 240, 360, 480, 720, 1,440, 2,880, and 4,320 min after administration. The blood samples were centrifuged at 5,000 rpm (4°C) for 7 min, and the supernatant plasma was harvested.

## Determination of UGT1A1 and Nine Other Transporter Proteins in the Liver

The detection method for UGT1A1 and nine other transporter proteins in the liver was comprehensively investigated in our study (35). We analyzed the different expression levels of bilirubin metabolizing enzyme and transporters in the liver of rats from the healthy animals, ANIT, and CCl_4_ model groups.

### Instrumentation and Chromatographic Conditions

An Agilent 1290 Infinity series UHPLC system coupled to an Agilent 6460 series MS/MS system (Agilent Technologies, Santa Clara, CA, United States) was applied to quantitate the signature peptides in ESI positive ionization mode. The mass spectrometry conditions were set up as follows: capillary voltage of 2000 V; gas flow at 8 L/min; nebulizer at 30 psi; gas temperature, 300°C; delta EMV (+) of 400. A 5 µl digest sample was injected into the column (Agilent SB-C18 column, 2.1 mm × 50 mm, 1.8 mm) and eluted at 0.4 ml/min with a gradient elution of water (with 0.05% v/v formic acid) (A) and acetonitrile (B) (0–1 min, 5–5% B; 1–4 min, 5–60% B; 4–5 min, 60–5% B; and re-equilibration for 3 min). The sequence of characteristic peptides that corresponded to UGT1A1 and the other nine transporter proteins were based on our previous experiments and listed in Table 3 **.**

**TABLE 3:** Multiple reaction monitoring parameters of peptides (and internal standard) selected for targeted analysis of hepatobiliary transporters.

Transports	Signature pedtides	Molecular weight	Parent ion (z = 2)	Product ion (z = 1)	Fragmentor	Collision energy
Mrp2	LTIIPQDPILFSGSLR	1770.08	885.7	1,329.9	200	25
Oct1	GVALPETIEEAENLGR	1,697.84	849.7	1,357.8	180	29
Ntcp	AAATEDATPAALEK	1,358.42	680	915.5	140	18
IS	AAATEDATPAALEK*	1,366.42	684	923	140	23
Oatp1a4	TFQFPGDIESSK	1,355.45	678.6	832.3	160	19
Mate1	LLLSGFQEELR	1,304.48	652.9	965	140	24
Bsep	STALQLIQR	1,029.19	515.5	529.5	130	17
Mdr1	NTTGALTTR	934.00	467.9	719.4	110	14
Oatp1a1	EENLGITK	903.00	452.3	468.1	120	8
Oatp1a2	SVQPELK	799.91	400.8	486.3	110	9
UGT1A1	TYPVPFQR	1,336.48	504.5	547.1	140	19

### Sample Preparation

Total membrane protein was isolated (in triplicate) from liver tissue samples according to the Native Membrane Protein Extraction Kit protocol. Subsequently, protein concentrations were determined by the BCA Protein Assay Kit. 10 µl of 5 mg/ml (or lower concentration) of a hepatocyte membrane protein incubated with 20 µl of dithiothreitol (100 mM) and 50 µl of ammonium bicarbonate buffer (50 mM, pH 7.8). After incubation at 95°C for 5 min, 20 µl of iodoacetamide (20 mM) was added to the mixture, followed by incubation at 37°C for 20 min in the dark. To concentrate the samples, ice-cold methanol (0.5 ml), chloroform (0.2 ml), and water (0.2 ml) were added. After centrifugation at 4°C for 5 min at 16,000 g, the supernatant was discarded and the pellet was washed once with ice-cold methanol (0.25 ml) and resuspended with 40 µl of reconstitution solution (equal volume of 3% sodium deoxycholate (w/v) and 5 mM ammonium bicarbonate buffer). Finally, the protein sample was digested with 10 µl of trypsin. The protein-to-trypsin ratio was 25:1 (w/w). After incubation at 37°C for 24 h, the digestion reaction was quenched by 60 µl of IS cocktail (prepared in 15% acetonitrile in water). The samples were centrifuged at 5,000 g for 5 min at 4°C, and 5 µl of the supernatant was injected in the UHPLC-MS/MS system.

### Data Processing and Statistical Analysis

All data were presented as mean ± standard deviation (SD). A one-way analysis of variance (ANOVA) was used to compare the data between three groups. The differences were considered to be statistically significant when *p* < 0.05 and highly significant when the *p-*value was <0.01 or *p* < 0.001. Spearman rank correlation coefficient, calculated by bivariate correlation analysis was used to evaluate the correlation between AUC values and expression levels of metabolic enzyme and transporters. All statistical analyses were performed with the SPSS Statistics system (SPSS vision 21.0). Pharmacokinetic data analyses were performed using DAS 2.1.1 software (Mathematical Pharmacology Professional Committee of China, Shanghai, China).

## Results

### Serum Biochemistry and Histopathological Examination

**FIGURE 3:**
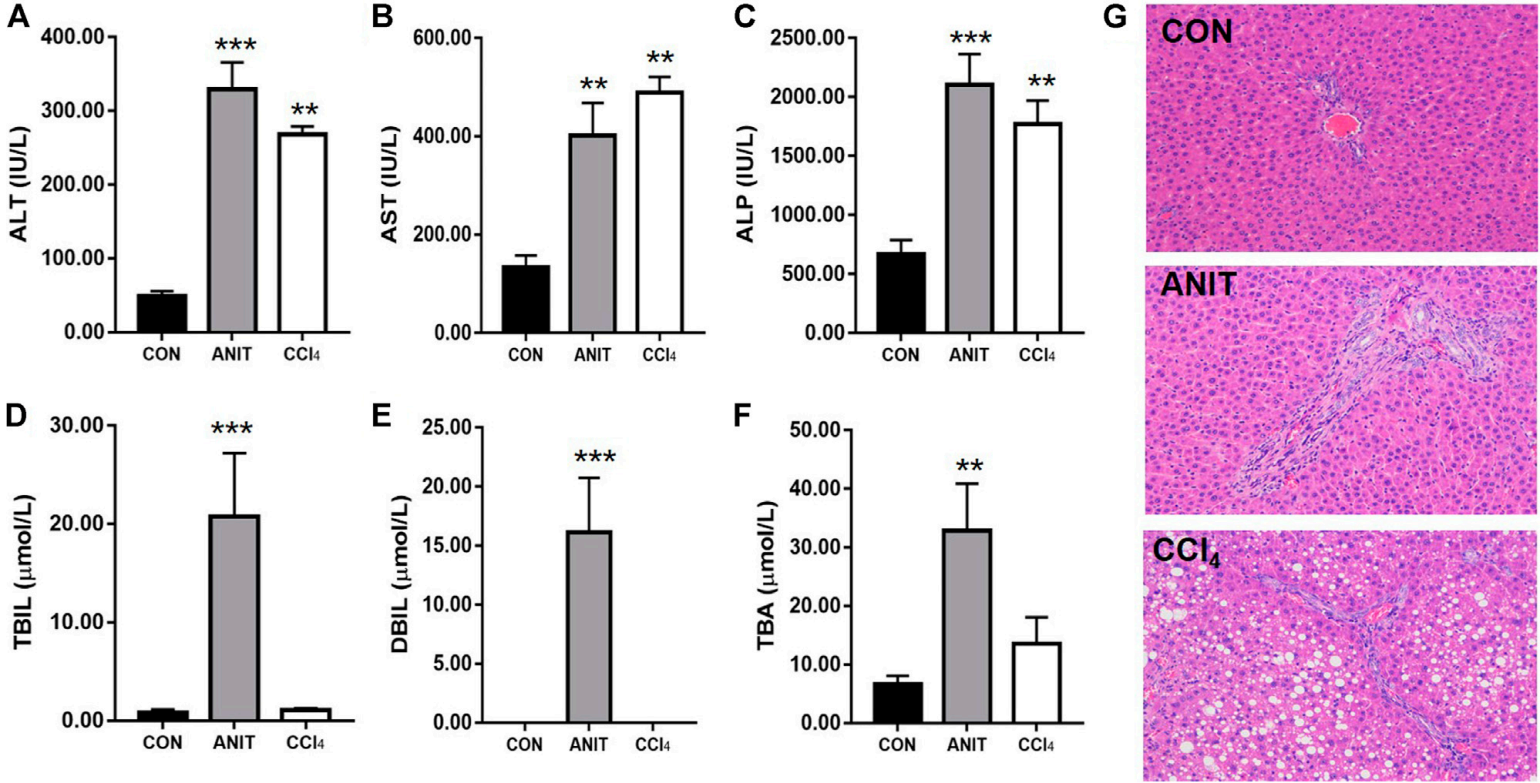
The results of serum biochemical and histopathological examination. **(A–F)** The serum ALT, AST, ALP, TBIL, DBIL, TBA, respectively. **(G)** Typical histopathological section photographs of rat liver speciments for H&E analysis ( ×40 magnification) (**p < 0.01 and ***p < 0.001 which compared with the control group).

The serum biochemical parameters such as TBIL, DBIL, ALT, and AST, were significantly increased in ANIT and CCl_4_ groups, and the livers also showed different pathological changes compared to the control group. In order to confirm the feasibility of our two pathological models, we examined the serum biochemical parameters and liver sections. The biochemistry serum results of ALT, AST, ALP, TBIL, DBIL, and TBA were presented in Figures 3A–F. ALT and AST are well-recognized markers of hepatocyte damage. As shown in Figures 3A,B the rats in group CCl_4_ dose efficiently increased the serum levels of ALT and AST to 4.45 and 2.68-folds (*p* < 0.001) respectively, compared with the control group, indicating that severe hepatocyte damage occurred after CCl_4_ administration. TBIL, DBIL, TBA and ALP were significantly increased in rats with cholestatic liver injury. As shown in Figures 3C–F, the rats in group ANIT dose efficiently increased the serum levels of TBIL, DBIL, TBA and ALP to 23.73, 1,612.00, 3.93 and 2.16-folds (*p* < 0.001) respectively, suggesting a severe cholestatic liver injury occurred after continuous administration of ANIT compared with the control group.

Liver sections from the healthy control group and the pathological model groups stained with hematoxylin and eosin were examined by microscopy to provide visual evidence. As shown in Figure 3G, the liver sections of the control animals showed normal hepatocyte structures. Marked cholestasis in terms of acute neutrophil infiltration, sinusoid congestion, and necrosis of the interlobular ducts and hepatocytes could be distinguished in specimens of ANIT-treated rats, compared with the livers from the healthy controls. The tissues from the CCl_4_ group showed disturbed hepatocyte arrangement, large number of hepatocytes with fatty degeneration in the form of vacuoles, few inflammatory infiltration and necrosis compared with the healthy control group. Two models of pathological liver injury were successfully established considering the results of serum biochemical and histopathological examination.

## Pharmacokinetic Studies of AE-PM in Different Rat Models

### Method Validation

#### Specificity

**FIGURE 4:**
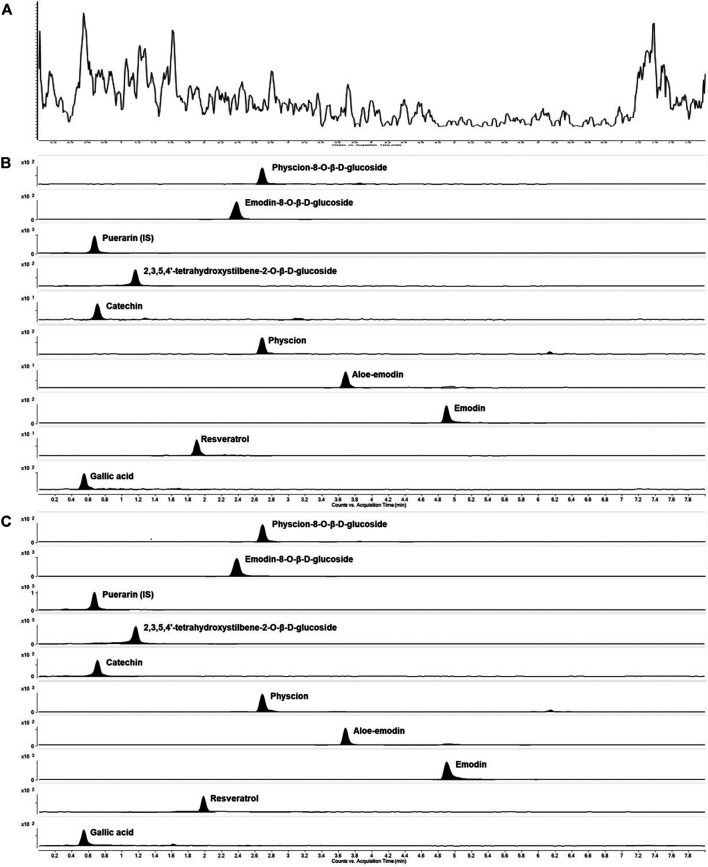
Representative MRM extracted chromatograms of PM and IS in rat plasma **(A)** a blank plasma sample **(B)** a blank plasma sample spiked with nine compounds at a low limit of quantification and IS **(C)** a plasma sample at 2 h after a single oral administration of AE-PM (50 g/kg).

The method selectivity was evaluated by comparing chromatograms of six extracted blank plasma samples of different sources with those of spiked plasma samples containing nine compounds and puerarin. Figures 4A,B shows the total ion chromatograms of the blank plasma sample and the QC samples spiked with nine components and puerarin by MRM scan, respectively. In addition, the real subject’s plasma sample total ion chromatograms collected at 60 min after the administration of AE-AM by oral gavage was presented in Figure 4C. Under optimal method conditions, no endogenous peaks of the analytes were observed in the retention time in any of the blank rat plasma batches, indicating that there was no significant endogenous interference of the MRM mode with the analytes during the assay.

#### Linearity

The calibration curves of the nine compounds were constructed respectively by plotting the peak-area ratio of each analyte to puerarin (y) vs each analyte concentration (ng/ml) (x) in spiked blank rat plasma. The method was linear over the concentration range of 7.64–29,650.00 ng/ml for TSG, 7.67–38,375.00 ng/ml for emodin, 5.92–5,800.00 ng/ml for aloe-emodin, 6.06–13,100.00 ng/ml for physcion, 6.01–12,700.00 ng/ml for E-Glu, 6.42–2,468.00 ng/ml for P-Glu, 6.66–2,560.00 ng/ml for gallic acid, 9.28–9,500.00 ng/ml for catechin, and 8.39–17,200.00 ng/ml for resveratrol. A signal noise ratio (S/N) ≥ 10 at the LLOQ was observed for all the analytes. In addition, it was observed that the LLOQ was7.64 ng/ml for TSG, 7.67 ng/ml for emodin, 5.92 ng/ml for aloe-emodin, 6.06 ng/ml for physcion, 6.01 ng/ml for E-Glu, 6.42 ng/ml for P-Glu, 6.66 ng/ml for gallic acid, 9.28 ng/ml for catechin, and 8.39 ng/ml for resveratrol. And the square of correlation coefficients (R^2^) was greater than 0.9811. The linear regression equations and correlation coefficients (R^2^) of the analytes are listed in Table 4.

**TABLE 4:** The results of linearity.

Component	Regression equation	*R* ^2^	Linearity range (ng/ml)	LLOQ (ng/ml)
TSG	Y = 0.006107*X+0.029365	0.9923	7.64–29,650.00	7.64
Emodin	Y = 0.026532*X+0.118,645	0.9987	7.67–38,375.00	7.67
Physcion	Y = 0.001945*X+0.064853	0.9901	6.06–13,100.00	6.06
Aloe-emodin	Y = 0.016824*X+0.032567	0.9972	5.92–5,800.00	5.92
Catechin	Y = 0.012869*X+0.06594	0.9900	9.28–9,500.00	9.28
E-glu	Y = 0.017420*X-0.055301	0.9933	6.01–12,700.00	6.01
P-glu	Y = 0.008064*X+0.024517	0.9910	6.42–2,468.00	6.42
Gallic acid	Y = 9.301,334*X+0.007394	0.9811	6.66–2,560.00	6.66
Resveratrol	Y = 0.000643*X+0.003861	0.9916	8.39–17,200.00	8.39

#### Accuracy and Precision

The accuracy and inter-day and intra-day precisions data at three concentrations of the nine analytes are listed in Table 5. Accuracy was calculated as RE% = (measured samples/spiked plasma-1) × 100%; the derived relative errors ranged from −9.8–16.2%**.** The inter-day and intra-day precision were in the ranges of 1.1–14.3 and 2.0–13.2% at all QC levels, respectively. The results showed that the method has good accuracy and precision, and was suitable for the pharmacokinetic analysis of all components.

**TABLE 5:** The results of accuracy and precision.

Component	Concentration (ng/ml)	Intra-day precision		Inter-day precision		Accuracy		
		Mean (ng/ml)	RSD (%)	Mean (ng/ml)	RSD (%)	Mean (ng/ml)	RE (%)	RSD (%)
TSG	14,825.00	14,876.26	4.1	15,023.17	4.2	15,981.35	7.8	2.3
	741.25	740.84	6.6	747.81	6.9	730.87	-1.4	4.1
	14.83	13.69	6.2	12.23	3.1	17.23	16.2	3.2
Emodin	38,375.00	38,406.12	6.9	37,697.78	6.3	19,590.44	2.1	5.9
	767.50	773.6	3.5	783.09	1.1	745.24	−2.9	7.7
	15.28	14.02	6.8	13.94	7.9	16.96	11.0	7.3
Aloe-emodin	2,900.00	3,012.06	9.3	2,987.48	3.6	2,876.80	−0.8	12.9
	362.50	356.72	4.1	325.67	2.2	362.14	−0.1	2.7
	12.79	11.64	6.3	10.44	11.1	12.78	−0.1	2.0
Physcion	6,550.00	6,618.43	3.3	6,667.23	2.9	7,015.05	7.1	4.2
	409.38	419.55	10.9	431.57	10.4	468.33	14.4	6.2
	12.79	13.65	13.2	10.49	14.3	13.35	4.4	1.5
E-glu	6,350.00	6,354.33	2.0	6,025.67	1.3	6,464.30	1.8	4.6
	396.88	396.16	4.2	405.48	4.3	359.97	−9.3	3.7
	12.40	11.22	5.5	12.19	6.1	13.22	6.6	2.8
P-glu	1,233.30	1,227.31	2.1	1,373.63	2.0	1,139.57	−7.6	3.3
	205.50	208.37	5.1	208.73	7.4	212.28	3.3	5.2
	12.84	12.42	3.9	12.92	2.9	13.69	6.6	13.9
Gallic acid	1,280.00	1,272.00	3.2	1,351.00	8.1	1,154.56	−9.8	10.2
	213.32	217.25	6.1	228.94	5.3	220.79	3.5	6.3
	13.32	15.64	5.1	12.82	6.8	14.47	8.6	2.6
Catechin	4,750.00	4,707.67	6.4	4,450.67	4.7	4,617.00	−2.8	5.3
	296.87	287.48	8.6	278.82	3.4	299.24	0.8	11.6
	18.56	18.06	3.4	17.9	8.9	20.94	12.8	8.4
Resveratrol	8,600.00	8,584.83	2.9	8,545.67	2.3	9,571.80	10.9	6.0
	537.50	550.09	2.6	569.86	1.7	539.65	−3.0	6.5
	16.78	17.50	5.8	17.00	6.7	15.49	−3.6	5.7

#### Extraction Recovery and Matrix Effect

The results of the matrix effect and extraction recovery of all components at three concentrations are shown in Table 6. The recovery range was from 48.9 to 104.6% at low, medium, and high concentrations for the nine constituents (RSD <14.5%), and the absolute matrix effect values ranged from 58.5 to 105.7%, with the RSD value being lower than 12.0%. The results indicated no coeluting peaks, which may have influenced the ionization of all components and puerarin.

**TABLE 6:** The results of matrix effect and recovery.

Component	Concentration (ng/ml)	Extraction recovery (%)		Matrix effect (%)	
		Mean (%)	RSD (%)	Mean (%)	RSD (%)
TSG	14,825.00	96.1	4.3	102.7	5.2
	741.25	81.7	5.2	96.6	3.8
	14.83	72.7	4.3	68.3	4.2
Emodin	38,375.00	102.7	4.0	102.7	5.9
	767.50	104.6	4.6	105.7	9.3
	15.28	99.3	2.6	99.7	1.1
Aloe-emodin	2,900.00	102.3	2.4	100.9	2.5
	362.50	82.4	3.3	103.2	9.3
	12.79	92.5	2.4	83.9	5.2
Physcion	6,550.00	102.1	3.3	98.5	1.4
	409.38	90.9	3.8	101.7	2.4
	12.79	80.0	7.2	91.0	4.1
E-glu	6,350.00	102.9	4.1	100.4	3.0
	396.88	99.8	3.1	101.2	5.6
	12.40	91.6	2.3	91.6	4.7
P-glu	1,233.30	94.8	3.1	99.6	4.8
	205.50	83.9	6.7	94.3	4.0
	12.84	79.5	7.6	82.4	5.4
Gallic acid	1,280.00	75.6	5.0	95.2	4.0
	213.32	65.2	4.3	76.2	5.2
	13.32	48.9	14.5	58.5	12.0
Catechin	4,750.00	50.5	6.3	99.0	2.5
	296.87	52	8.4	102.4	6.8
	18.56	50.7	14.3	77	8.1
Resveratrol	8,600.00	93.9	4.3	99.1	1.4
	537.50	74.4	3.8	98	1.2
	16.78	72.1	3.7	72.9	8.0

#### Stability

The stability of QC samples of the nine compounds were tested under three different conditions (Table 7). All compounds were shown to be stable (RSD ranged from 1.7 to 12.6%) in rat plasma at room temperature for 24 h. After extraction, all analytes were found to be stable (RSD range from 1.3 to 13.8%) in the reconstitution solution at 4°C for 24 h. Besides, all compounds were shown to be stable (RSD ranged from 1.7 to 14.9%) for three freeze-thaw cycles in rat plasma. In our experiment, it was also observed that the unprocessed QC samples were stable (RSD range from 1.5 to 13.9%) for 60 days when stored at −80°C. The data indicated that the nine compounds in plasma were all stable for 24 h at room temperature, three freeze/thaw cycles, 24 h at 4°C, and for 60 days when stored at −80°C.

**TABLE 7:** The results of stability.

Component	Concentration (ng/ml)	Room temperature 24 h(ng/mL)	RSD (%)	4°C24h (ng/ml)	RSD (%)	Freeze-thaw (ng/ml)	RSD (%)	Long stability (ng/ml)	RSD (%)
TSG	14,825.00	14,588.47	1.9	14,393	9.7	13,676.34	5.0	14,032.10	4.2
	741.25	707.81	5.1	735.65	1.3	799.25	3.2	735.67	9.0
	14.83	12.23	4.0	18.27	6.4	15.70	8.4	16.57	10.1
Emodin	38,375.00	18,338.88	7.9	19,254.57	5.0	16,020.76	7.2	19,872.84	11.6
	767.50	783.9	6.7	773.62	2.4	703.82	11.0	723.65	9.1
	15.28	15.38	3.8	12.28	7.0	17.00	9.1	12.72	5.3
Aloe-emodin	2,900.00	2,867.01	6.1	3,021.55	2.6	3,101.50	1.7	2,756.34	7.8
	362.50	375.62	10.1	325.67	9.9	408.33	1.8	357.26	8.5
	12.79	11.44	12.6	10.24	8.0	9.68	5.6	13.55	2.7
Physcion	6,550.00	6,258.35	10.0	6,618.34	8.4	6,534.22	6.3	6,896.46	11.3
	409.38	431.57	4.2	429.55	13.7	395.96	7.5	401.67	4.3
	12.79	12.42	3.4	15.65	1.9	9.78	7.7	13.77	10.2
E-glu	6,350.00	6,025.12	12.5	2054.33	11.8	6,202.46	7.5	6,435.66	13.9
	396.88	366.48	8.7	366.16	2.6	409.91	10.8	394.13	12.3
	12.40	13.19	6.1	15.22	9.3	11.32	11.4	11.58	11.9
P-glu	1,233.30	1,129.33	7.8	1,238.43	11.0	1,309.48	14.9	1,286.72	4.4
	205.50	221.06	9.2	208.37	7.8	197.10	13.8	200.84	5.3
	12.84	15.64	1.7	12.92	2.2	12.02	11.7	13.58	12.3
Gallic acid	1,280.00	1,342.38	11.9	1,272.02	13.8	1,188.50	12.9	1,306.35	7.9
	213.32	278.94	4.7	277.25	12.2	199.66	7.6	201.39	8.3
	13.32	13.56	11.5	12.82	8.9	11.38	4.9	14.12	6.6
Catechin	4,750.00	4,650.67	4.6	4,654.36	11.6	4,999.10	11.6	4,726.31	6.7
	296.87	301.23	6.4	277.48	9.3	302.41	7.0	286.34	10.6
	18.56	18.06	9.7	17.90	4.2	18.6	2.9	20.9	5.2
Resveratrol	8,600.00	8,738.41	6.8	8,654.18	1.4	8,399.20	11.5	8,513.48	2.4
	537.50	521.81	4.4	542.29	1.9	501.81	6.6	514.25	1.5
	16.78	16.28	8.8	15.64	8.8	18.84	7.3	15.34	6.7

### Pharmacokinetics Study on the Major Components and Phase II Metabolites

**FIGURE 5:**
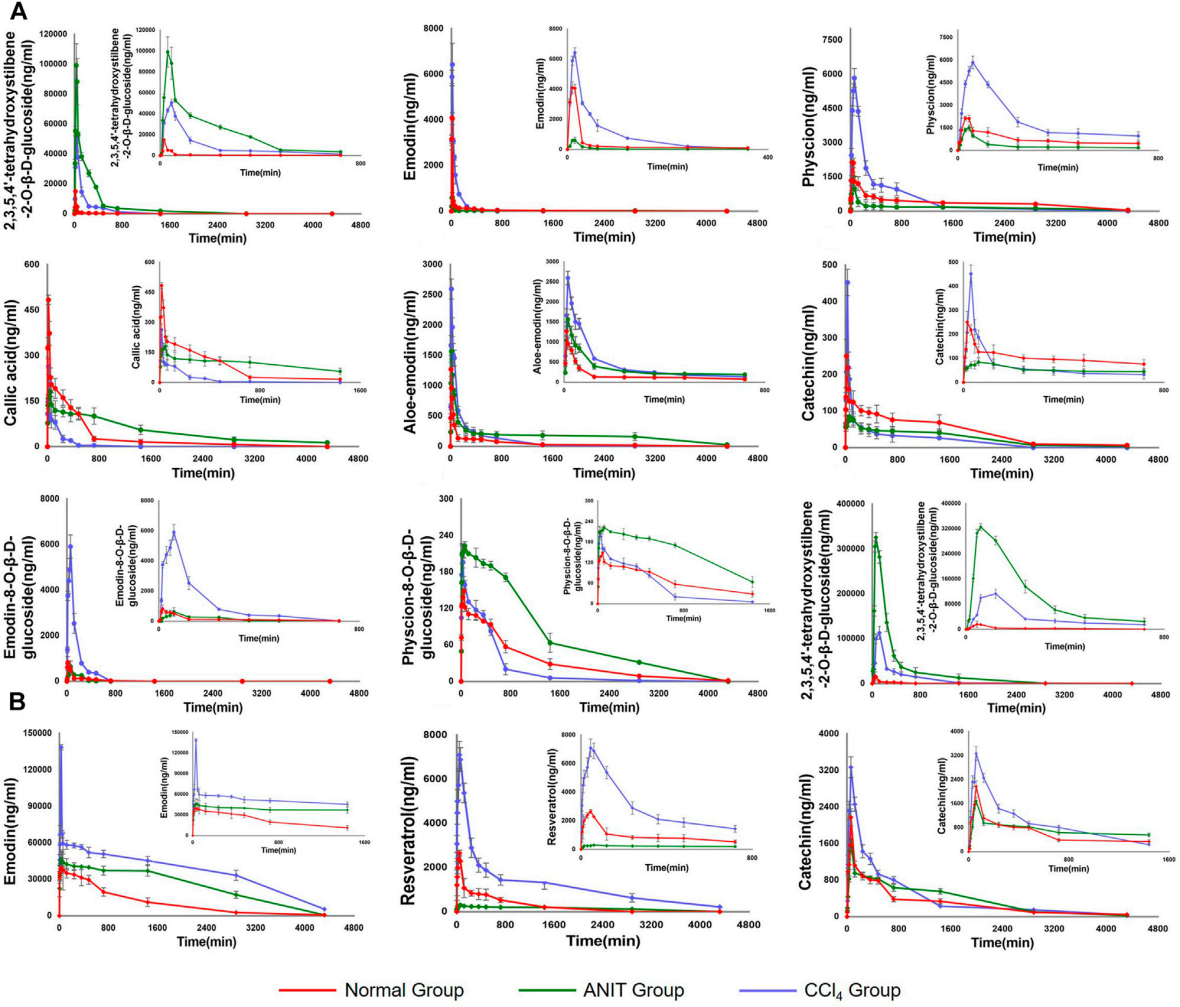
**(A)** The pharmacokinetic curves of each components in normal, ANIT and CCl_4_ group of rats, which were treated with AE-PM at dose of 50 g/kg i. g. and the serums were not treated with sulfatase. **(B)** The pharmacokinetic curves of phase II metabolites of TSG, emodin, resveratrol and catechin, respectively. The data was the difference between the content of post-enzymolysis and pre-enzymolysis.

Following the administration of AW-PM, eight prototype components (TSG, emodin, physcion, E-Glu, P-Glu, aloe-emodin, catechin, and gallic acid) and five phase II metabolites (TSG, emodin, catechin, and physcion) could be detected by UPLC-MS/MS method. Resveratrol has been fully converted into a phase II metabolite and could not be detected directly. The mean plasma concentration-time profiles of TSG, emodin, physcion, aloe-emodin, callic acid, catechin, E-Glu, and P-Glu in normal, cholestatic and hepatocytes damaged rats are shown in Figure 5A **.** TSG, emodin and catechin had been partially converted into phase II metabolites, as shown in Figure 5B. The pharmacokinetic behaviors of the majority of PM components were significantly different in the ANIT and CCl_4_ model rats when compared with the control group. The pharmacokinetic data are shown in Table 8.

**TABLE 8:** Correlation results of AUC values with metabolic enzymes and transporters (**p < 0.01, *p < 0.05).

Component	Correlation coefficient ρ									
	UGT1A1	BSEP	OATP1A4	OCT1	OATP1A2	NTCP	MRP2	MDR1	OATP1A1	MATE1
TSG-II	−0.832**	−0.854**	−0.539*	−0.879**	−0.461	−0.864**	−0.757**	−0.886**	−0.836**	−0.332
Emodin-II	−0.504	−0.479	−0.834**	−0.296	0.371	−0.189	−0.236	−0.446	−0.279	0.361
Resveratrol-II	0.418	0.357	−0.109	0.489	0.718**	0.579*	0.529*	0.450	0.496	0.825**
Catechin-II	−0.436	−0.489	−0.741**	−0.229	0.286	−0.146	−0.157	−0.375	−0.254	0.207
TSG	−0.875**	−0.932**	−0.573*	0.900**	−0.489	−0.804**	−0.771**	−0.911**	−0.911**	−0.436
Emodin	0.411	0.361	−0.082	0.439	0.675**	0.557*	0.557*	0.479	0.604*	0.832**
E-glu	0.186	0.129	−0.204	0.179	0.571*	0.468	0.282	0.218	0.361	0.625*
Physcion	0.386	0.293	-0.247	0.457	0.714**	0.554*	0.539*	0.379	0.514*	0.668**
P-glu	−0.354	−0.300	0.181	−0.486	−0.736**	−0.536*	−0.593*	−0.475	−0.532*	−0.789**
Aloe-emodin	−0.914**	−0.825**	−0.702**	−0.807**	−0.400	−0.754**	−0.800**	−0.936**	−0.786**	−0.504
Catechin	0.250	0.264	0.560*	0.164	−0.336	−0.079	0.179	0.193	−0.018	−0.561*
Gallic acid	−0.439	−0.354	0.145	−0.446	−0.757**	−0.525*	−0.518*	−0.443	−0.568*	−0.850**

Compared with the normal group, the AUC (0-∞) and Cmax values of TSG in the ANIT model increased by 2,548.17% and 560.12%, respectively, and 802.14 and 236.59% respectively in the CCl_4_ model. The AUC (0-∞) values of emodin, physcion and E-Glu increased by 75.69, 29.24, and 655.90% respectively in CCl_4_ model group, and the Cmax values increased by 57.02, 173.72, and 620.15% respectively. While in the ANIT model, the AUC (0-∞) values of emodin, physcion, and E-Glu decreased by 55.98, 49.77 and 8.25%, respectively, the Cmax values decreased by 85.18, 29.48, and 22.98%, respectively.

As the common drugs contain −OH, −COOH, and −SH groups, the primary way of metabolism *in vivo* is to combine these radicals with endogenous α-D-glucuronide to produce β - glucuronide. PM mainly contains stilbene glycosides, anthraquinones, tannins and other components, as shown in Figure 1, which cintain multiple -OH radicals. For example, TSG (Sun, 2004; Lv et al., 2011b) and emodin are reported to metabolize glucuronidation *in vivo*. The metabolic pathway of these compounds are related to the metabolic pathway in bilirubin, which may affect the key enzymes and transporters of bilirubin metabolism (Yi et al., 2018). Therefore, it is essential to study these metabolites metabolism *in vivo*. Accordingly, we used biological enzymolysis combined with UHPLC-MS/MS technology to detect these compounds. After sulfatase-aided enzymolysis, all the phase II metabolites could transform into their prototypes. The specific contents of the phase II metabolites of these components were detected indirectly by evaluating the content difference of their prototypes in samples before and after enzymolysis. By considering the difference in the content of their prototypes in those conditions, we can indirectly determine whether these components had phase II metabolism and the specific content.

The pharmacokinetic curves of phase II metabolites are illustrated in Figure 5B. Resveratrol was immediately and completely transformed into phase II metabolites. After sulfatase-aided enzymolysis, the AUC(0-∞) and Cmax values in CCl_4_ group were significantly higher than that in normal group (*p* < 0.05), while its metabolism in ANIT group was significantly inhibited. The trends of AUC(0-∞) and Cmax of TSG in different models were consistent with the prototype, and emodin exhibited a similar pattern. The AUC(0-∞) and Cmax values of II phase metabolite of TSG were the highest in the ANIT model, and increased by 2,943.73% and 1917.18% respectively compared with the normal group; the AUC(0-∞) and Cmax values of emodin II phase metabolites were the highest in the CCl_4_ group, and increased by 522.45 and 250.27%, respectively, when compared with the normal group.

### Metabolic Enzyme and Transporters Expression in Rat Liver

**FIGURE 6:**
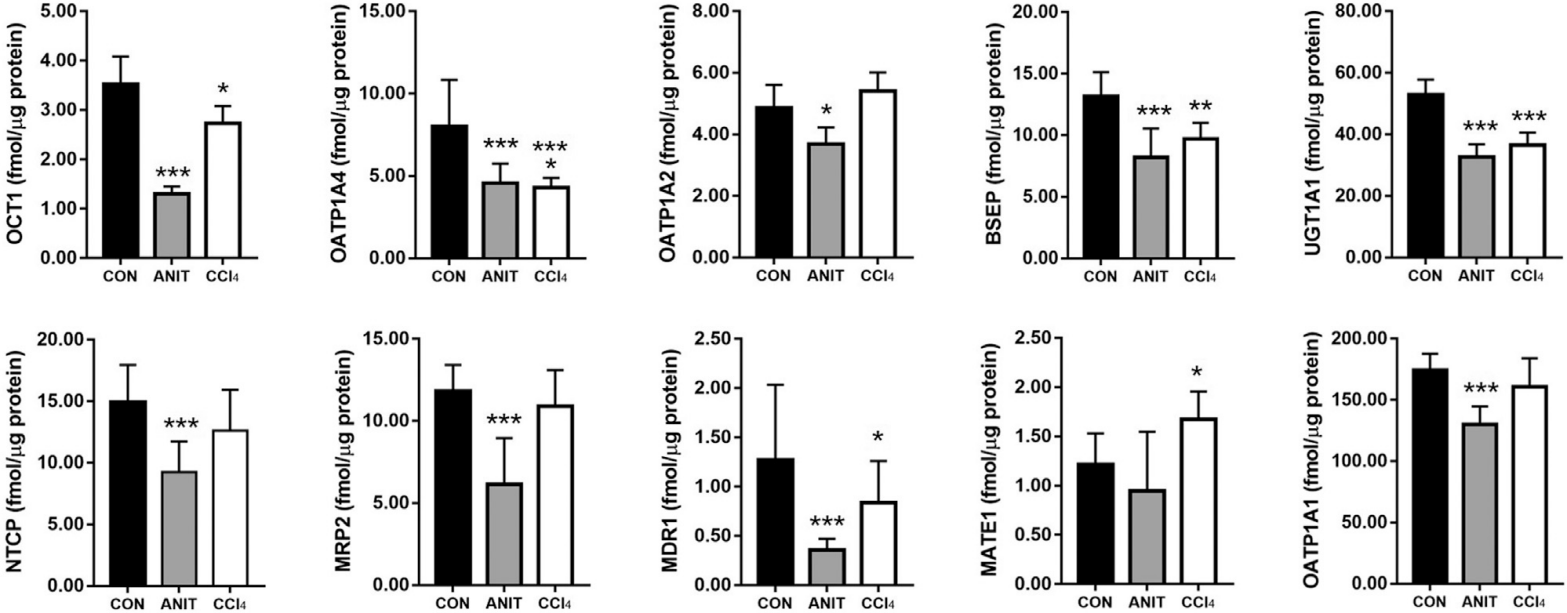
The expression levels of metabolic enzyme UGT1A1 and nine transporters NTCP, OATP1A1, OATP1A2, OATP1A4, MDR1, BSEP, OCT1, MATE1, MRP2 in rat liver, respectively (*p < 0.05, **p < 0.01 and ***p < 0.001 which compared with the control group).

The expression levels of metabolic enzyme and transporters in the rat livers were used to evaluate the liver status of rats in different pathological models. The quantitative results of the metabolic enzyme UGT1A1 and the nine transporters (MRP2, BSEP, OCT1, NTCP, MATE1, MDR1, OATP1A1, OATP1A2, and OATP1A4) are listed in Figure 6. The downregulation of BSEP and MRP2 expression is a major indicator of cholestasis (Yi et al., 2018). Before administration of AE-PM, the BSEP and MRP2 expression levels in ANIT group were significantly reduced by 38.15 and 47.90%, respectively, when compared with the control group, indicating that ANIT-induced cholestasis had been successfully established in this pathological experiment. Furthermore, UGT1A1, OCT1, NTCP, MDR1, OATP1A2 and OATP1A4 of this model group decreased significantly by 38.22, 62.92, 38.02, 72.37%, 24.28, and 42.58% (*p* < 0.05), respectively, when compared with the control group. Although the mechanism of CCl_4_-induced liver injury was significantly different from that of ANIT, it also showed a more obvious inhibitory effect on the expression levels of liver metabolic enzymes and transporters. Compared with the control group, the expression levels of UGT1A1, BSEP, OATP1A4, OCT1, NTCP and MDR1 were reduced by 31.18, 26.92, 46.11, 22.30, 15.75, and 34.15%, respectively (*p* < 0.05).

### Correlation Between Area Under The Curve Values and Expression Levels of Metabolic Enzyme and Transporters

The area under the curve (AUC) is an important indicator to evaluate the degree of drug absorption, reflecting the exposure characteristics of the drug *in vivo*. The critical enzymes and transporters located on the hepatocyte membrane are involved in the uptake, transformation and excretion of endogenous or exogenous substances by the liver. To further investigate the reasons for the differences in the pharmacokinetics of PM in different liver pathological models, we made a correlation analysis between the AUC values of the active ingredients in PM and the expression levels of metabolic enzymes and transporters in the liver. The results are shown in Table 9, Supplementary Figures S1-S54.

**TABLE 9:** Pharmacokinetic parameters of PM after oral administration (*n* = 6) at the doses of 50 g/kg. Data were expressed as mean ± SD.

	TSG			Emodin		
	Normal group	ANIT group	CCl_4_ group	Normal group	ANIT group	CCl_4_ group
AUC (0-∞) (ug/L*h)	12,860.002 ± 1,267.111	340,555.228 ± 8,676.096^***^	116,021.02 ± 3,800.387^***^	3,396.707 ± 266.27	1,495.195 ± 99.774^***^	5,967.825 ± 451.127^***^
MRT (0-∞) (h)	4.54 ± 1.225	6.031 ± 1.926^**^	3.4 ± 1.039^**^	6.093 ± 1.83	22.849 ± 5.28^***^	1.234 ± 0.681^***^
t1/2z (h)	1.947 ± 0.806	2.606 ± 0.612^**^	2.806 ± 0.878^*^	3.681 ± 1.075	3.913 ± 0.821	3.654 ± 0.267
Tmax (h)	0.25 ± 0.0293	0.5 ± 0.0278^***^	0.75 ± 0.136^***^	0.167 ± 0.059	0.25 ± 0.063^**^	0.25 ± 0.0659^**^
Vz/F (L/kg)	4.369 ± 0.711	0.221 ± 0.0206^***^	0.698 ± 0.073^***^	31.274 ± 3.246	75.533 ± 8.863^***^	68.125 ± 6.027^***^
CLz/F (L/h/kg)	1.555 ± 0.15	0.059 ± 0.008^***^	0.172 ± 0.0484^***^	5.888 ± 1.3	13.376 ± 1.245^***^	3.351 ± 2.464^**^
Cmax (ug/L)	15,042.977 ± 1,420.87	99,300.972 ± 1,092.408^***^	50,633.759 ± 2,885.149^***^	4,095.622 ± 119.478	606.742 ± 29.949^***^	6,430.985 ± 552.673^***^
	**E-glu**			**Catechin**		
		**Normal group**	**ANIT group**	**CCl4 group**	**Normal group**	**ANIT group**	**CCl4 group**
AUC (0-∞) (ug/L*h)	1878.279 ± 537.5	1723.354 ± 169.583	14,197.913 ± 845.631^***^	12,627.797 ± 498.523	11,568.084 ± 195.158^**^	1,502.738 ± 140.965^***^
MRT (0-∞) (h)	3.881 ± 1.519	2.813 ± 0.872^***^	2.437 ± 1.173^***^	145.555 ± 11.305	252.563 ± 3.754^***^	11.218 ± 1.227^***^
t1/2z (h)	3.92 ± 0.219	4.678 ± 1.101^**^	4.002 ± 1.399	103.734 ± 14.95	177.313 ± 1.093^**^	3.757 ± 0.816^**^
Tmax (h)	0.25 ± 0.0301	1 ± 0.248^***^	1 ± 0.289^***^	0.25 ± 0.0234	1 ± 0.742^***^	0.5 ± 0.0408^***^
Vz/F (L/kg)	60.229 ± 9.446	78.332 ± 4.397^***^	8.135 ± 1.747^***^	237.077 ± 18.348	442.359 ± 16.868^***^	72.16 ± 12.592^***^
CLz/F (L/h/kg)	10.648 ± 4.718	11.605 ± 2.029	1.409 ± 0.24^***^	1.584 ± 0.27	1.729 ± 0.229	13.309 ± 1.754^***^
Cmax (ug/L)	821.44925 ± 84.984	632.694 ± 49.957^***^	5,915.695 ± 871.195^***^	251.047 ± 11.768	84.472 ± 8.848^***^	452.775 ± 8.941^***^
	**Physcion**			**P-glu**		
		**Normal group**	**ANIT group**	**CCl_4_ group**	**Normal group**	**ANIT group**	**CCl_4_ group**
AUC (0-∞) (ug/L*h)	27,822.335 ± 773.899	13,974.221 ± 624.579^***^	35,958.959 ± 528.665^***^	2,271.749 ± 78.37	7,052.215 ± 315.03^***^	1,449.713 ± 82.063^***^
MRT (0-∞) (h)	26.749 ± 1.523	37.577 ± 2.271^**^	10.042 ± 1.144^***^	15.929 ± 2.36	18.829 ± 3.363	7.483 ± 1.42^**^
t1/2z (h)	16.979 ± 1.869	28.937 ± 1.502^***^	8.78 ± 1.604^***^	11.252 ± 1.851	3.224 ± 0.473^***^	4.085 ± 0.453^***^
Tmax (h)	0.5 ± 0.094	0.75 ± 0.0238^**^	1 ± 0.0675^***^	0.75 ± 0.049	1 ± 0.232^**^	0.25 ± 0.0661^***^
Vz/F (L/kg)	17.612 ± 2.411	59.761 ± 3.407^***^	7.047 ± 1.106^***^	142.94 ± 11.136	13.195 ± 1.861^***^	81.316 ± 5.01^**^
CLz/F (L/h/kg)	0.719 ± 0.0478	1.431 ± 0.494^***^	0.556 ± 0.085^*^	8.804 ± 0.88	2.836 ± 0.596^***^	13.796 ± 0.503^**^
Cmax (ug/L)	2,135.037 ± 55.906	1,505.553 ± 16.353^***^	5,844.122 ± 36.939^***^	148.403 ± 8.946	222.994 ± 13.243^**^	211.025 ± 3.041^**^
	**Aloe-emodin**			**Gallic acid**		
		**Normal group**	**ANIT group**	**CCl_4_ group**	**Normal group**	**ANIT group**	**CCl_4_ group**
AUC (0-∞) (ug/L*h)	3,563.013 ± 34.244	41,745.244 ± 693.83^***^	6,619.038 ± 344.087^***^	2,774.695 ± 59.062	6,502.744 ± 59.616^***^	436.565 ± 9.708^***^
MRT (0-∞) (h)	13.988 ± 1.42	198.2 ± 3.166^***^	6.469 ± 0.422^**^	12.834 ± 1.023	55.689 ± 4.221^***^	3.064 ± 0.12^***^
t1/2z (h)	3.545 ± 0.185	139.843 ± 8.294^***^	3.448 ± 0.177	19.71 ± 0.891	37.733 ± 4.056^**^	4.695 ± 0.509^**^
Tmax (h)	0.167 ± 0.051	0.25 ± 0.032^**^	0.25 ± 0.052^**^	0.25 ± 0.0728	0.75 ± 0.0231^***^	0.25 ± 0.0641
Vz/F (L/kg)	28.711 ± 1.55	96.678 ± 8.349^***^	15.032 ± 1.204^*^	205.009 ± 2.203	167.465 ± 6.197^**^	310.403 ± 9.634^***^
CLz/F (L/h/kg)	5.613 ± 0.385	0.479 ± 0.074^***^	3.022 ± 0.826^**^	7.208 ± 0.841	3.076 ± 0.35^***^	45.812 ± 2.249^***^
Cmax (ug/L)	1,272.720 ± 70.28	1,569.070 ± 47.023	2,598.319 ± 41.816^***^	484.264 ± 9.872	181.248 ± 5.001^***^	263.718 ± 2.794^**^
	**Phase II metabolites of TSG**			**Phase II metabolites of emodin**		
		**Normal group**	**ANIT group**	**CCl_4_ group**	**Normal group**	**ANIT group**	**CCl_4_ group**
AUC (0-∞) (ug/L*h)	56,190.799 ± 289.468	1,710,295.269 ± 2,609.142^***^	606,652.048 ± 4,198.509^***^	772,114.586 ± 1,634.327	1,809,403.474 ± 9,951.471^***^	4,805,993.415 ± 9,226.949^***^
MRT (0-∞) (h)	11.038 ± 1.186	7.645 ± 0.169^**^	8.185 ± 1.344^**^	16.848 ± 1.376	23.806 ± 1.097^**^	84.003 ± 2.207^***^
t1/2z (h)	16.558 ± 1.827	9.698 ± 1.212^**^	8.044 ± 0.349^***^	12.383 ± 0.714	9.807 ± 1.319^*^	58.469 ± 1.646^***^
Tmax (h)	0.75 ± 0.089	1 ± 0.443^*^	2 ± 0.513^**^	0.5 ± 0.516	0.5 ± 0.053	0.5 ± 0.043
Vz/F (L/kg)	8.505 ± 1.824	0.164 ± 0.055^***^	0.383 ± 0.029^***^	0.463 ± 0.028	0.156 ± 0.047^**^	0.351 ± 0.087^*^
CLz/F (L/h/kg)	0.356 ± 0.0329	0.012 ± 0.003^***^	0.033 ± 0.006^***^	0.026 ± 0.002	0.011 ± 0.003^**^	0.004 ± 0.001^***^
Cmax (ug/L)	16,148.939 ± 250.383	325,752.767 ± 6,950.049^***^	113,483.559 ± 6,667.748^***^	39,590.057 ± 217.721	46,655.793 ± 393.253^**^	138,673.836 ± 8,681.865^***^
	**Phase II metabolites of resveratrol**			**Phase II metabolites of catechin**		
		**Normal group**	**ANIT group**	**CCl_4_ group**	**Normal group**	**ANIT group**	**CCl_4_ group**
AUC (0-∞) (ug/L*h)	18,593.476 ± 932.739	10,375.617 ± 995.877^**^	91,942.212 ± 533.694^***^	23,072.119 ± 618.38	27,634.972 ± 736.603^*^	30,882.787 ± 700.99^**^
MRT (0-∞) (h)	9.847 ± 1.347	24.184 ± 2.355^***^	25.348 ± 1.77^***^	22.223 ± 2.708	19.423 ± 1.143^*^	16.855 ± 1.062^*^
t1/2z (h)	3.014 ± 0.049	3.175 ± 0.98	18.283 ± 3.832^***^	20.127 ± 2.371	13.171 ± 1.493^**^	13.797 ± 2.022^**^
Tmax (h)	0.75 ± 0.086	1 ± 0.297^*^	0.75 ± 0.015	1 ± 0.175	1 ± 0.089	1 ± 0.239
Vz/F (L/kg)	4.678 ± 0.823	8.831 ± 0.838^***^	5.739 ± 0.852^*^	25.176 ± 1.472	13.754 ± 1.303^***^	12.894 ± 1.439^***^
CLz/F (L/h/kg)	1.076 ± 0.173	1.928 ± 0.328^*^	0.218 ± 0.018^***^	0.867 ± 0.089	0.724 ± 0.016^*^	0.648 ± 0.052^*^
Cmax (ug/L)	2,658.743 ± 41.707	306.1102 ± 9.416^***^	7,112.017 ± 63.531^***^	2,174.588 ± 55.393	1,682.419 ± 29.805^**^	3,271.904 ± 42.486^***^

ANOVA test was used to calculate the significance of the differences, ***p < 0.001, **p < 0.01, and *p < 0.05 which compared with the Normal group.

The expression levels of UGT1A1 (*ρ* = −0.832, ρ = −0.875, ρ = −0.914), BSEP (ρ = −0.854, ρ = −0.932, ρ = −0.825), OCT1 (ρ = −0.879, ρ = −0.900, ρ = −0.807) and MDR1 (ρ = −0.886, ρ = −0.911, ρ = −0.936) (*p* < 0.01) were significantly correlated with the exposure characteristics of TSG prototype and its phase II metabolites and aloe-emodin *in vivo*, respectively. In addition, the expression levels of NTCP, MRP2 and OATP1A1 were found in relation to the AUC values of TSG prototype (ρ = −0.804, ρ = −0.771, ρ = −0.911) and its phase II metabolites (ρ = −0.864, ρ = −0.757, ρ = −0.836), aloe-emodin (ρ = −0.754, ρ = −0.800, ρ = −0.786) (*p* < 0.01), emodin (ρ = 0.557,ρ = 0.557,ρ = 0.604), physcion (ρ = 0.554, ρ = 0.539, ρ = 0.514), P-Glu (ρ = -0.536, ρ = −0.593, ρ = −0.532) and gallic acid (ρ = -0.525, ρ = −0.518, ρ = −0.568) (*p* < 0.05), respectively. And the expression levels of OATP1A2 and MATE1 significantly correlated with the AUC values of phase II metabolites of resveratrol (ρ = 0.718, ρ = 0.825), emodin (ρ = 0.675, ρ = 0.832), physcion (ρ = 0.714, ρ = 0.668), P-Glu (ρ = −0.736, ρ = −0.789), gallic acid (ρ = −0.757, ρ = −0.850) (*p* < 0.01) and E-Glu (ρ = 0.571, ρ = −0.625) (*p* < 0.05). Besides, the expression levels of OATP1A4 were significantly correlated with the AUC values of aloe-emodin (ρ = −0.702), the phase II metabolites of emodin (ρ = −0.834) (*p* < 0.01), the prototypes of TSG (ρ = −0.573) and catechin (ρ = 0.560) (*p* < 0.05) and their phase II metabolites (ρ = −0.539, ρ = −0.741) (*p* < 0.01).

## Discussion

PM-induced liver injury is limited by the cholestatic type, but also includes hepatocellular injury (Lianhong et al., 2015; Duan et al., 2020; Yamakawa et al., 2020). In the present study, ANIT and CCl_4_ were used as models of clinical liver injury. ANIT is a common inducer of intrahepatic cholestasis experimental models and induce cholestasis by destroying the bile duct epithelial cells and hepatocytes. Bile duct obstruction, severe apoptosis or necrosis of interlobular duct epithelial cells are characteristics of ANIT, and most prominent 24–48 h after model induction. TBIL and DBIL in the serum were significantly increased, the expression levels of the bilirubin metabolizing enzymes UGT1A1 and transporters OATP2, MRP2 and BSEP were notably suppressed (Li et al., 2016; Yi et al., 2018). CCl_4_ is a chemical inducer of liver injury, mainly through the formation of free radicals, triggering chain reactions of peroxidation induced by hepatocyte damage (Shuwen and Zhen, 2019). It is featured by coagulative necrosis in the central region of the hepatic lobules and vacuolar degeneration in the surrounding hepatocytes (Xiaohui et al., 2018), with markedly elevated expression levels of ALT and AST (Lianhong et al., 2015). The results of serum biochemical and histopathological examinations showed that the models of ANIT and CCl_4_ were successfully achieved.

The pharmacokinetic characteristics of the nine components of AE-PM were studied by using UPLC-MS/MS. The sulfatase hydrolysis technique was used for the indirect determination of phase II metabolites of TSG, emodin, resveratrol and catechins. Physcion also undergoes glucuronidation and sulfation, consistent with the results of the Zhang J.’ study (Zhang et al., 2018a), but the levels of conjugates were too low to allow pharmacokinetic analysis. Prototype of resveratrol cannot be detected in all three groups before hydrolysis, indicating that it is mainly presented in glucuronic acid and sulfuric acid conjugation. Resveratrol was not detected in the plasma of PM (before and after concoction) in Zhang L.’ study (Zhang et al., 2013). 81.36, 83.39, and 83.95% TSG, 99.56, 99.92, and 99.88% emodin, 64.63, 70.49, and 95.36% catechin exist in normal, ANIT and CCl_4_ groups in the form of the phase II metabolites. The AUC(0-∞) values of phase II metabolite of resveratrol in control, ANIT, and CCl_4_ groups were 18,593.48, 10,375.62, and 91,942.21 μg/L, respectively. The conversion rates of phase II metabolites of TSG and emodin among the three groups did not present significant differences. However, overall, the exposure levels of phase II metabolites of TSG and emodin were higher in both pathological models. Catechins and resveratrol were more inclined to undergo phase II metabolism in the CCl_4_ model. However, the expression level of UGT1A1 involved in phase II metabolism was significantly inhibited in ANIT and CCl_4_ models. It is possible that the drug might be more susceptible to phase II metabolism due to the compensatory increase of UGT1A1 in both pathological models.

The Tmax value of phase II metabolite of TSG in CCl_4_ models was 120 min, to the other components in the different groups were less than 60 min. Aloe-emodin, emodin prototype and its II phase metabolite presented Tmax values smaller than 30 min, indicating that these components absorbed rapidly. The AUC(0-∞) and Cmax values of the TSG prototype and its phase II metabolites were higher in the ANIT model, followed by CCl_4_ group, and the normal group. These two components had higher exposure and slower elimination in both pathological states. The AUC(0-∞) and Cmax values of emodin, physcion, phase II metabolites of emodin, and resveratrol were higher in the CCl_4_ model, followed by the normal group and the ANIT group. The liver cell damage caused by CCl_4_ would promote their absorption into the blood and inhibit its elimination, while cholestasis could hinder the absorption. Aloe-emodin had higher exposure and slower elimination in ANIT group. Catechins and P-Glu could be rapidly absorbed and eliminated in the CCl_4_ model, but cholestasis could inhibit their elimination. The absorption and elimination of gallic acid could be inhibited in the case of cholestasis. The phase II metabolite of catechin could be absorbed and eliminated rapidly in the normal group but inhibited in ANIT and CCl_4_. The exposure levels of physcion, emodin, E-Glu, phase II metabolites of emodin and catechin were higher in CCl_4_-induced pathological state. In contrast TSG, aloe-emodin, gallic acid and the phase II metabolite of TSG were higher in the cholestasis state.

There was a correlation between the exposure characteristics of the active ingredients in PM and the expression levels of liver metabolizing enzymes and transporters, with TSG and aloe-emodin being the most pronounced. The AUC values of TSG and aloe-emodin showed a significant negative correlation with the expression levels of UGT1A1, BSEP, OATP1A4, OCT1, NTCP, MRP2, and MDR1. This result suggests that the metabolisms of TSG and aloe-emodin by the liver were weakened when metabolic enzymes and transporters were inhibited, resulting in the elevated occurrence of TSG and aloe-emodin in the blood. The AUC values of emodin, physcion, and resveratrol were positively correlated with the expression levels of OATP1A2, NTCP, and MRP2, suggesting that the excretions of emodin, physcion and resveratrol were promoted when the expression levels of OATP1A2, NTCP, and MRP2 were reduced. Besides, the expression levels of UGT1A1 decreased in normal rats, while the expression levels of UGT1A1, MATE1, BSEP, and OCT1 increased in the ANIT and CCl_4_ groups after administration of AE-PM for 72 h, compared with those when AE-PM was not administered. Our results might suggest that PM could upregulate the expression levels of bilirubin metabolizing enzymes and transporters in injured liver and downregulate them in the normal liver. Finally, the mechanism of hepatic metabolizing enzymes and transporters that affect the metabolism of the active ingredients in PM needs further investigation.

## Conclusion

A more comprehensive description of the pharmacokinetics of PM was investigated. The phase II metabolites TSG, emodin, resveratrol and catechin were preliminarily analyzed. In addition, a priliminary correlation analysis of the expression levels of hepatic metabolizing enzymes and transporters with the pharmacokinetic parameters of PM was done in this work. At the same time, the specific mechanisms of the interaction between these two variables that affect each other still needs further experimental investigation.

## Data Availability

The original contributions presented in the study are included in the article/Supplementary Material, further inquiries can be directed to the corresponding authors.

## Abbreviations

PM, *Polygonum multiflorum* Thunb; AE-PM, alcohol extracts of *Polygonum multiflorum* Thunb; UPLC-MS/MS, ultra-high performance liquid chromatography-tandem mass spectrometry; TSG, 2,3,5,4'-tetrahydroxystilbene-2-O-β-D-glucoside; E-Glu, emodin-8-O-β-D-glucoside; P-Glu, physcion-8-O-β-D-glucoside; ANIT, alpha-naphthylisothiocyanate; CCl_4_, Carbon tetrachloride.
