# Supplementary material for: Comparative Studies on Multi-Component Pharmacokinetics of Polygonum multiflorum Thunb Extract After Oral Administration in Different Rat Models
Source: Front Pharmacol. 2021 Jun 17;12:655332. doi: 10.3389/fphar.2021.655332 (PMC8245786; doi:10.3389/fphar.2021.655332)
Supplement: Supplementary file 1 [file DataSheet1.docx]

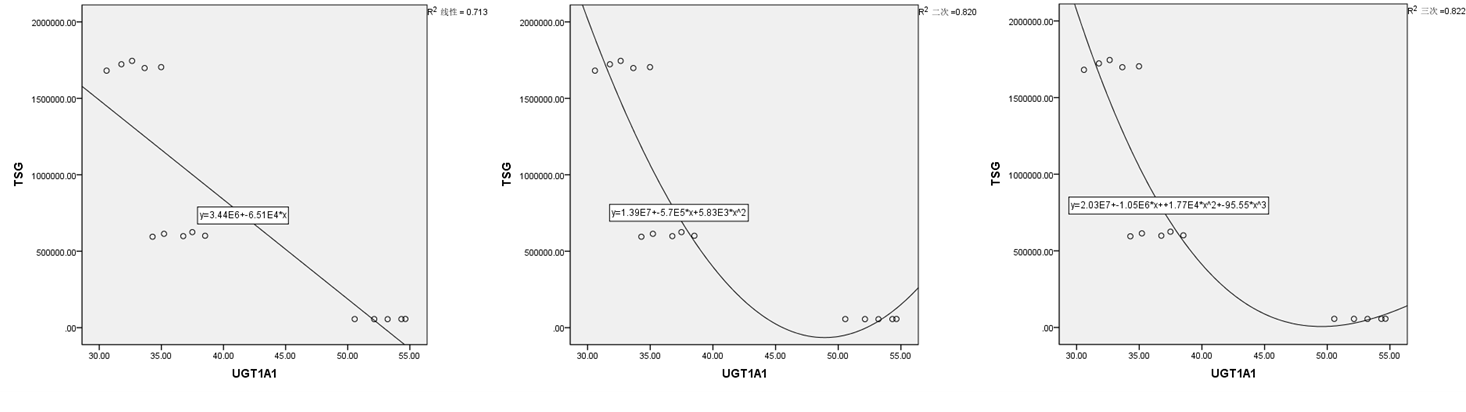


**Supplementary Figure 1** The correlation between the expression level of UGT1A1 and the AUC of phase II metabolites of TSG (*p*<0.01)


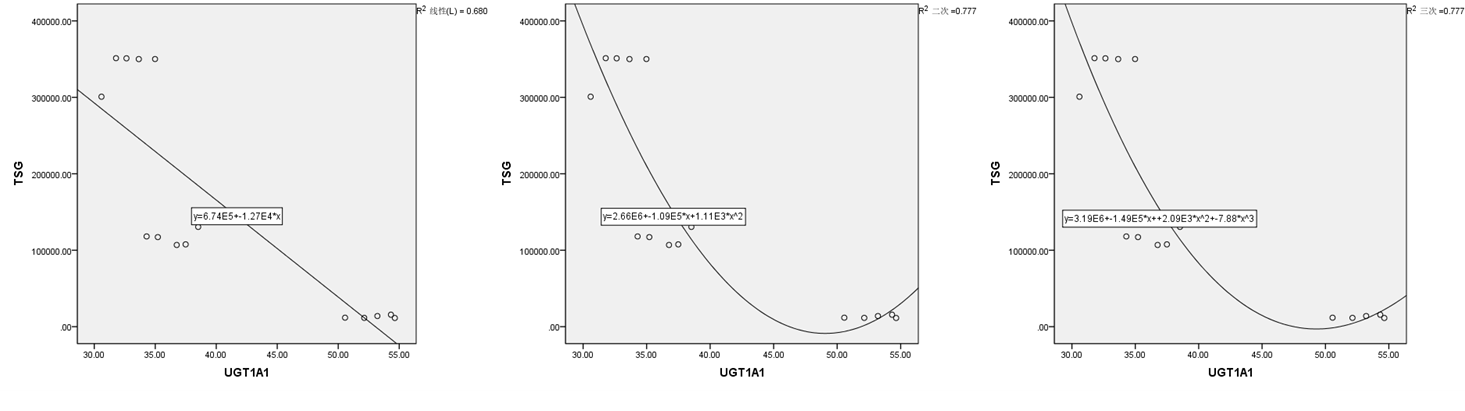


**Supplementary Figure 2** The correlation between the expression level of UGT1A1 and the AUC of TSG (*p*<0.01)


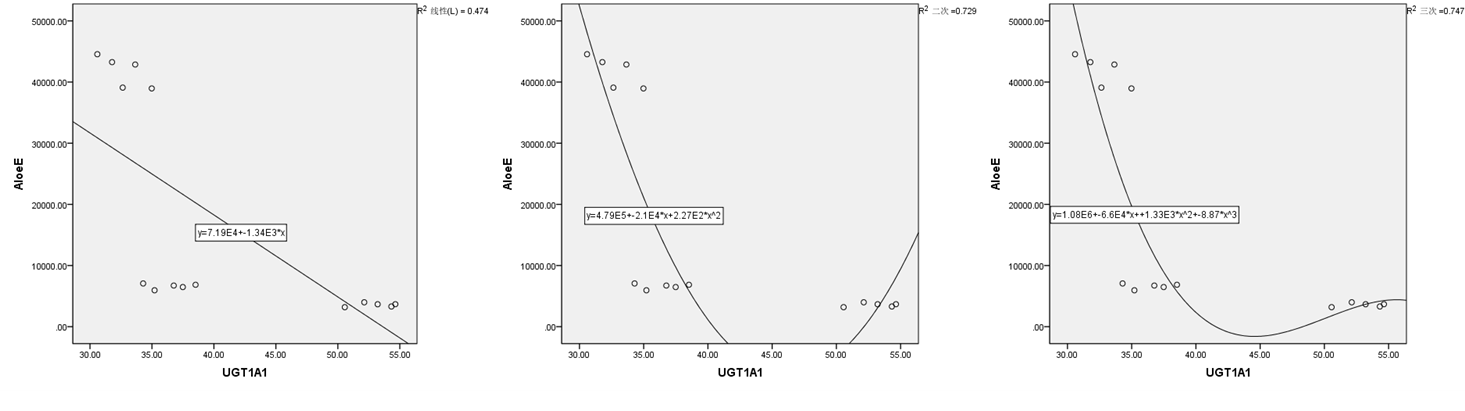
**Supplementary Figure 3** The correlation between the expression level of UGT1A1 and the AUC of aloe-emodin (*p*<0.01)


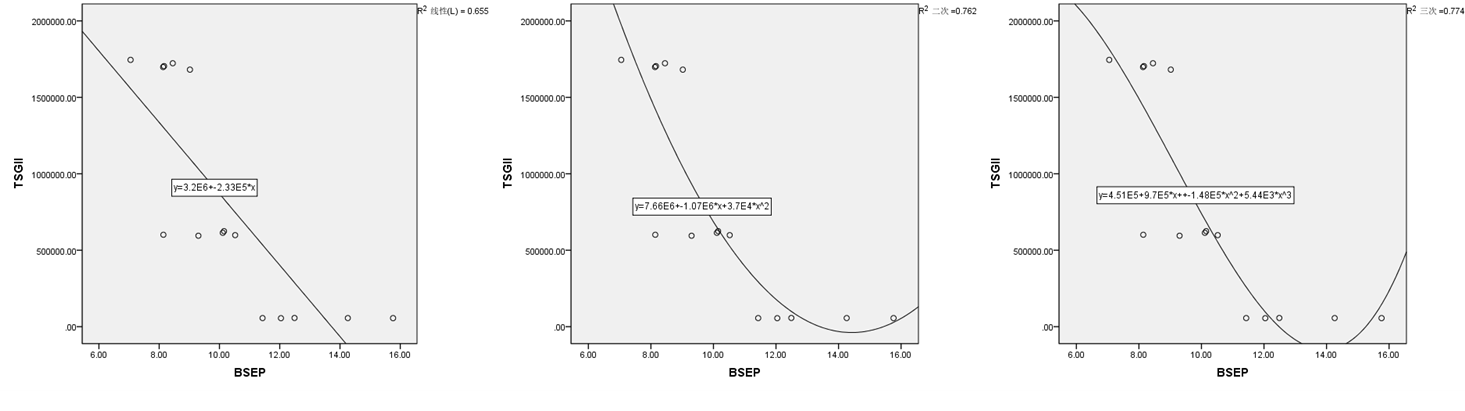
**Supplementary Figure 4** The correlation between the expression level of BSEP and the AUC of phase II metabolites of TSG (*p*<0.01)


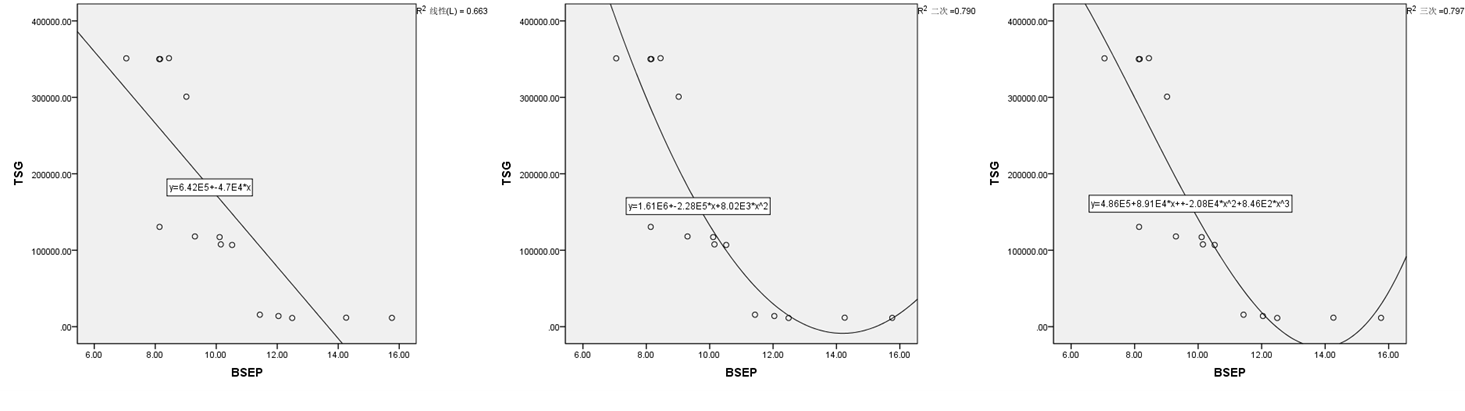
**Supplementary Figure 5** The correlation between the expression level of BSEP and the AUC of TSG (*p*<0.01)


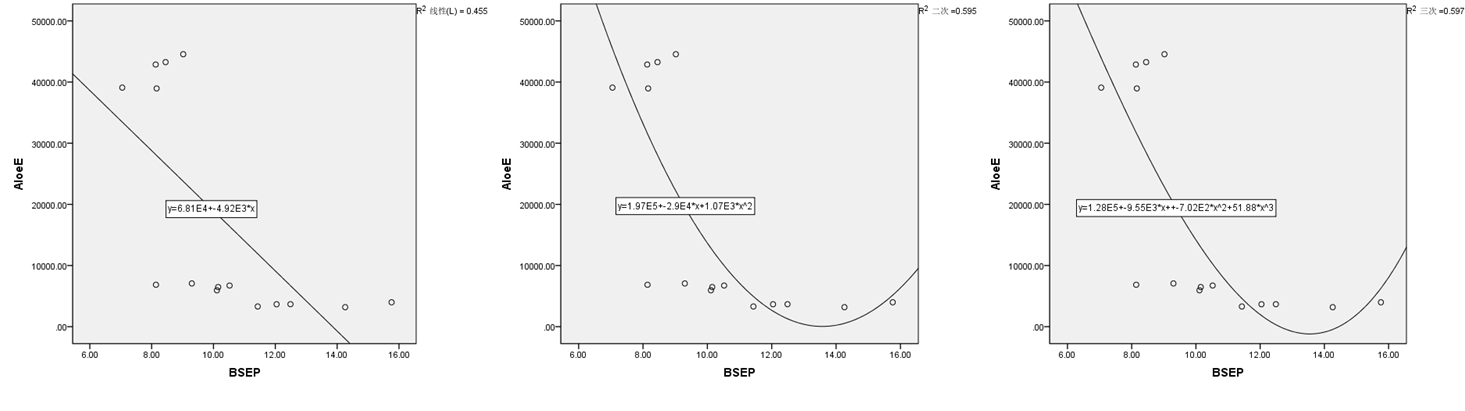


**Supplementary Figure 6** The correlation between the expression level of BSEP and the AUC of aloe-emodin (*p*<0.01)


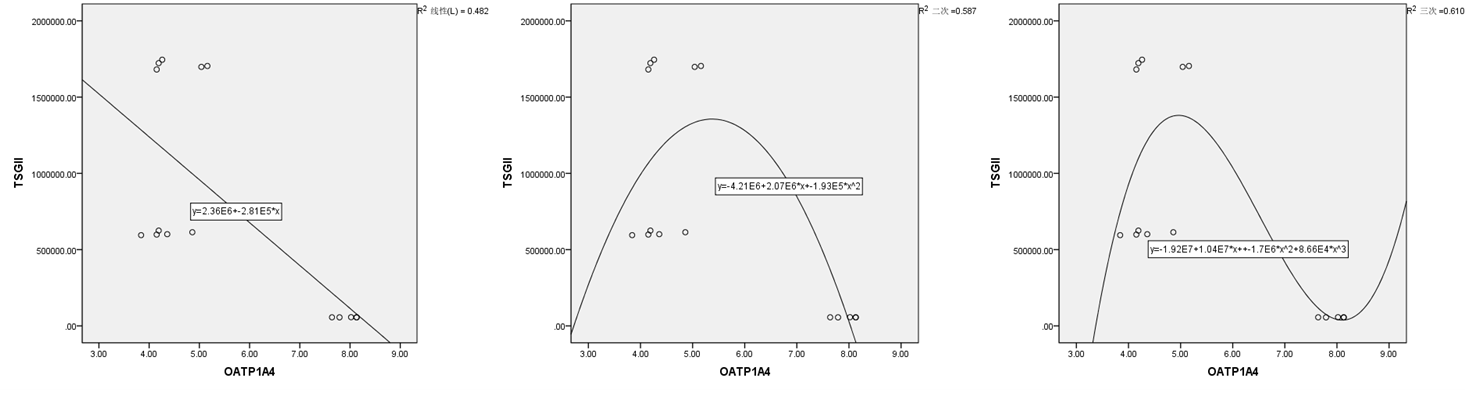


**Supplementary Figure 7** The correlation between the expression level of OATP1A4 and the AUC of phase II metabolites of TSG (*p*<0.01)


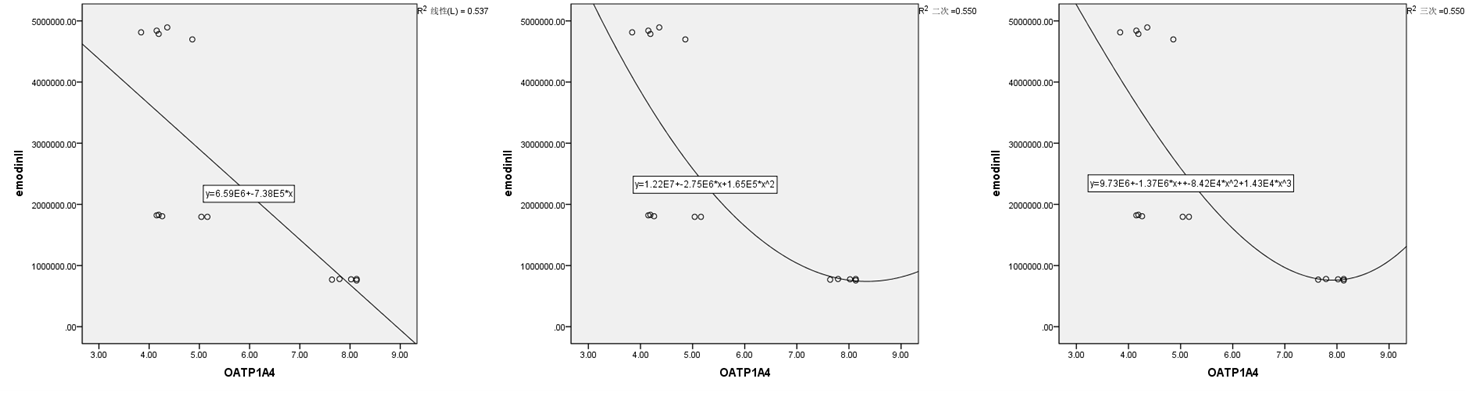


**Supplementary Figure 8** The correlation between the expression level of OATP1A4 and the AUC of phase II metabolites of emodin (*p*<0.01)


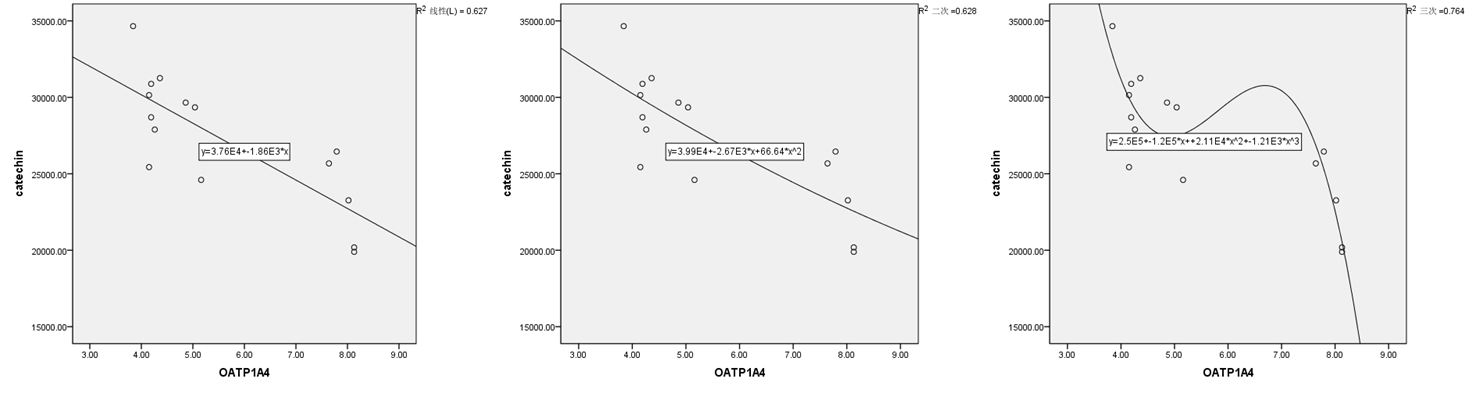


**Supplementary Figure 9** The correlation between the expression level of OATP1A4 and the AUC of phase II metabolites of catechin (*p*<0.01)


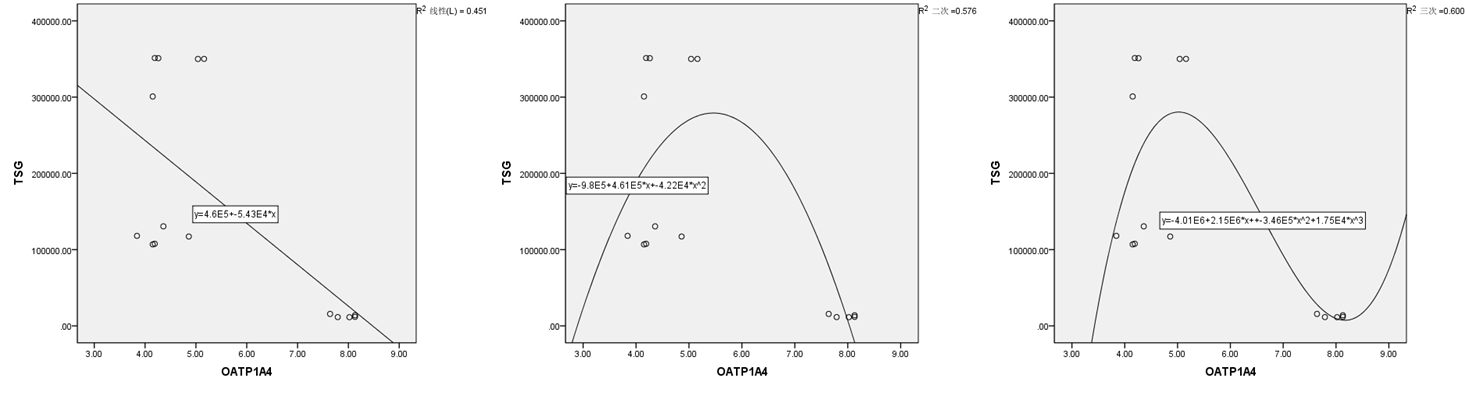


**Supplementary Figure 10** The correlation between the expression level of OATP1A4 and the AUC of TSG (*p*<0.05)


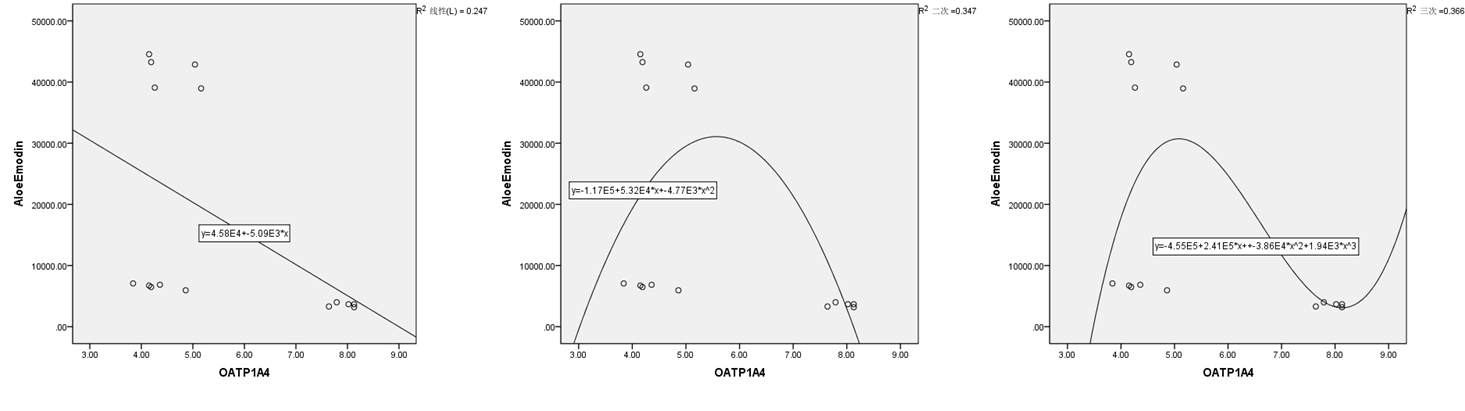


**Supplementary Figure 11** The correlation between the expression level of OATP1A4 and the AUC of aloe-emodin (*p*<0.01)


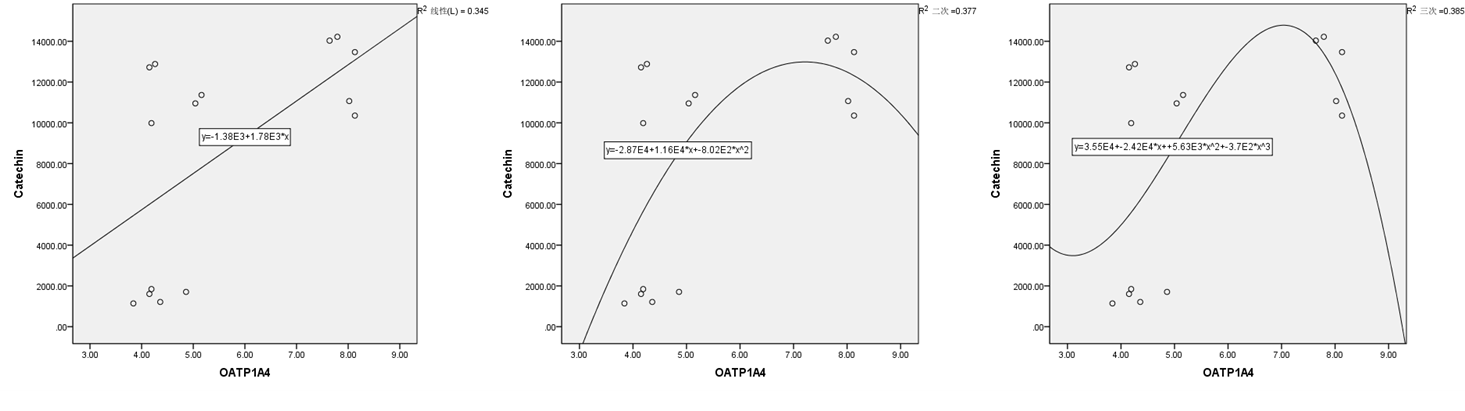


**Supplementary Figure 12** The correlation between the expression level of OATP1A4 and the AUC of catechin (*p*<0.05)


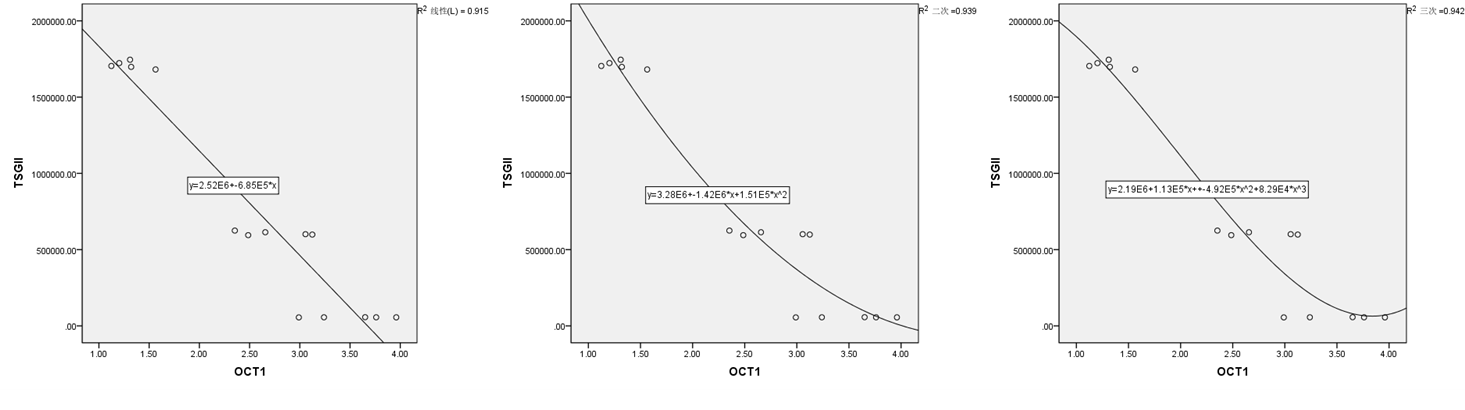


**Supplementary Figure 13** The correlation between the expression level of OCT1 and the AUC of phase II metabolites of TSG (*p*<0.01)


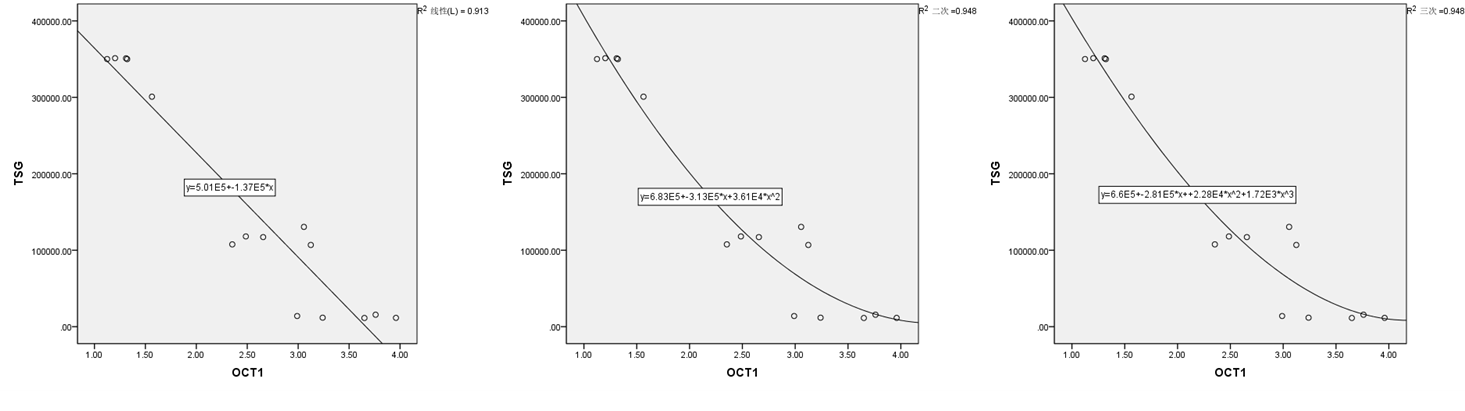


**Supplementary Figure 14**The correlation between the expression level of OCT1 and the AUC of TSG (*p*<0.01)


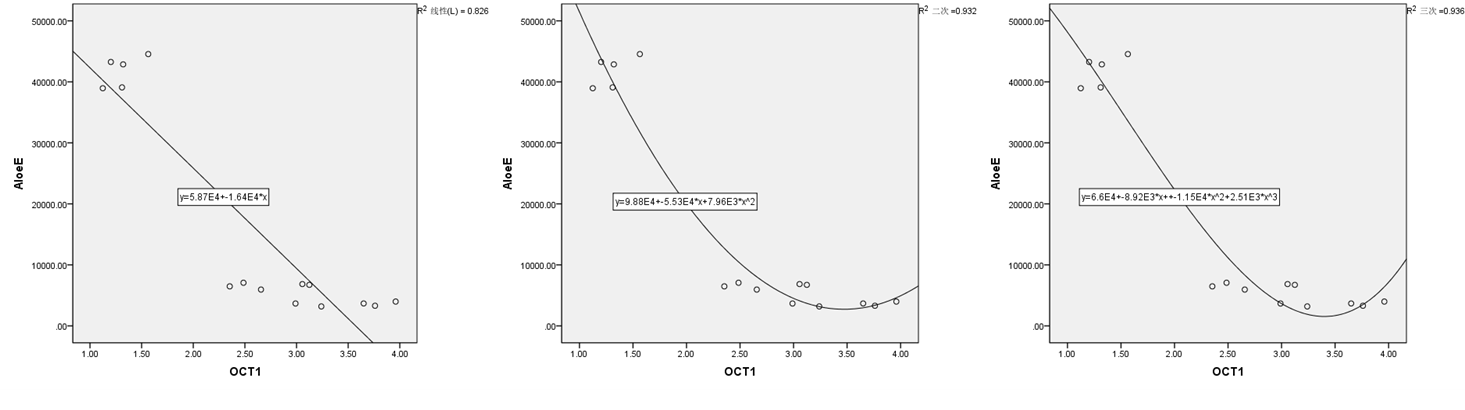


**Supplementary Figure 15** The correlation between the expression level of OCT1 and the AUC of aloe-emodin (*p*<0.01)


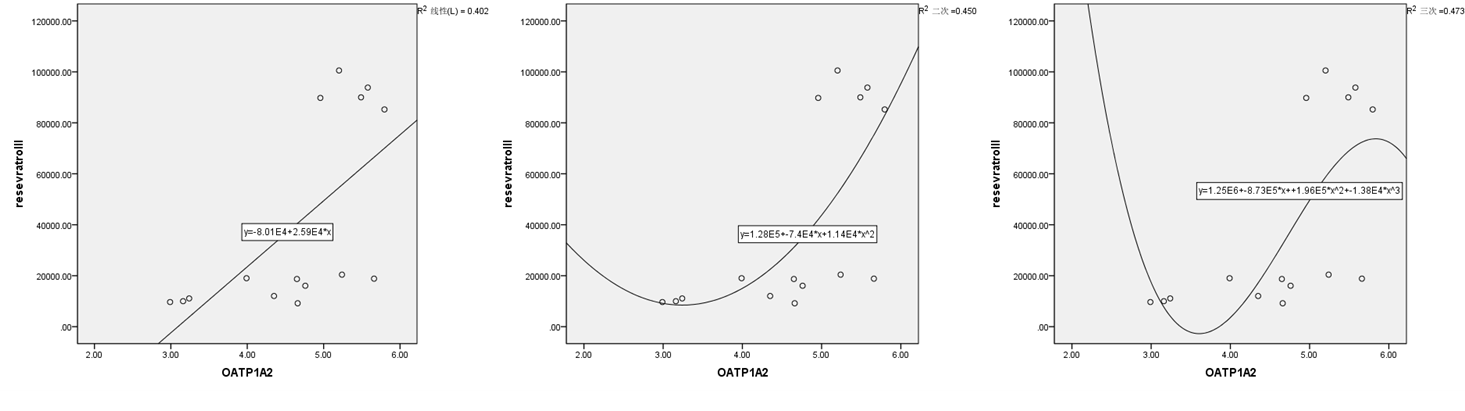


**Supplementary Figure 16** The correlation between the expression level of OATP1A2 and the AUC of phase II metabolites of resveratrol (*p*<0.01)


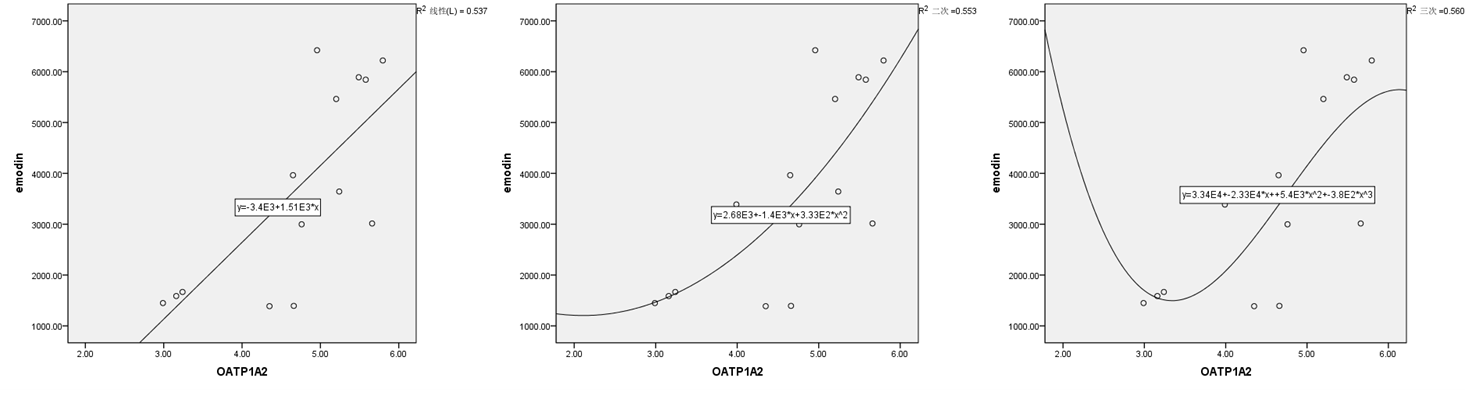


**Supplementary Figure 17** The correlation between the expression level of OATP1A2 and the AUC of emodin (*p*<0.01)


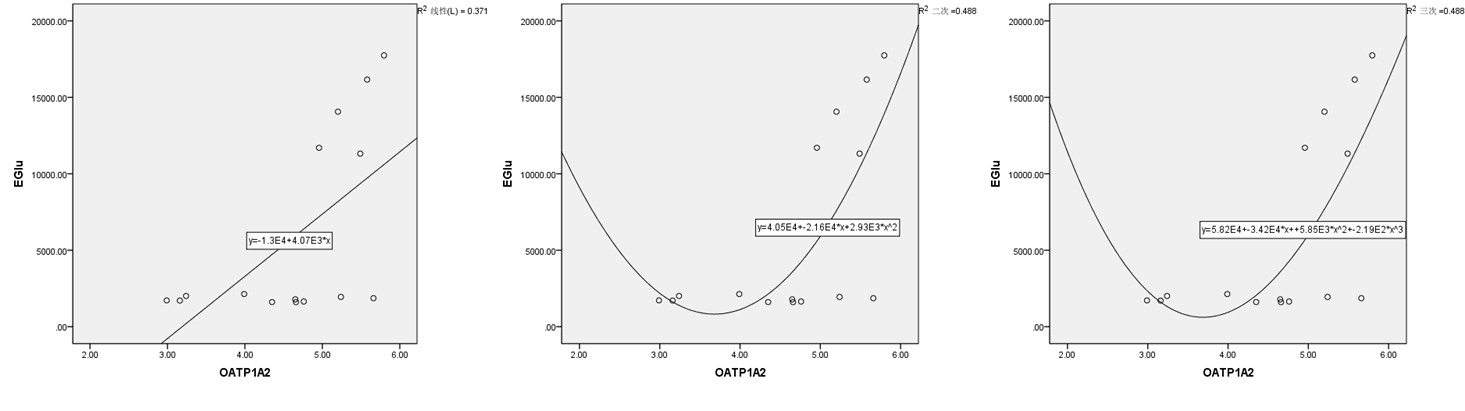


**Supplementary Figure 18** The correlation between the expression level of OATP1A2 and the AUC of E-Glu (*p*<0.05)


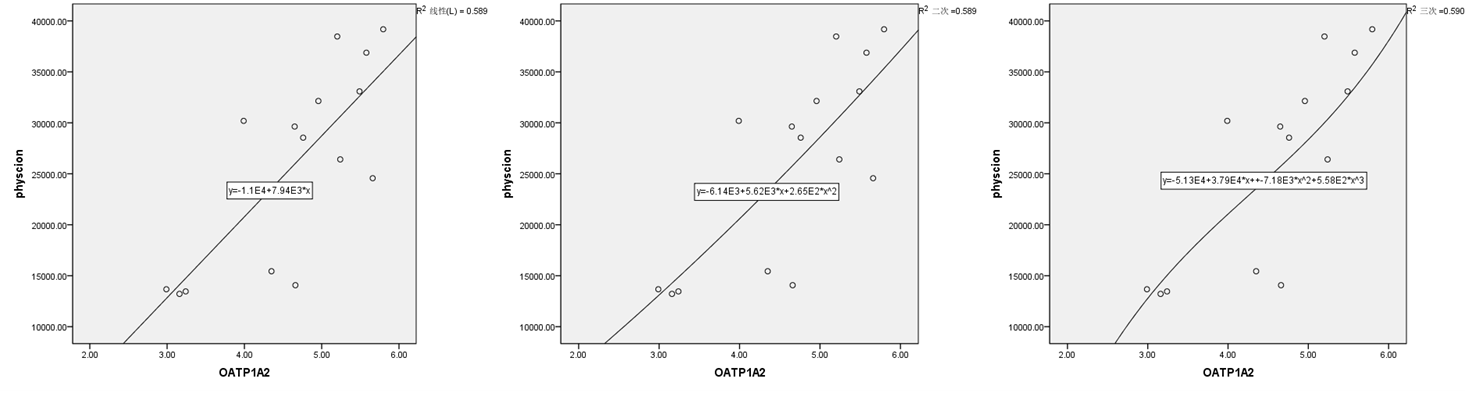


**Supplementary Figure 19** The correlation between the expression level of OATP1A2 and the AUC of physcion (*p*<0.01)


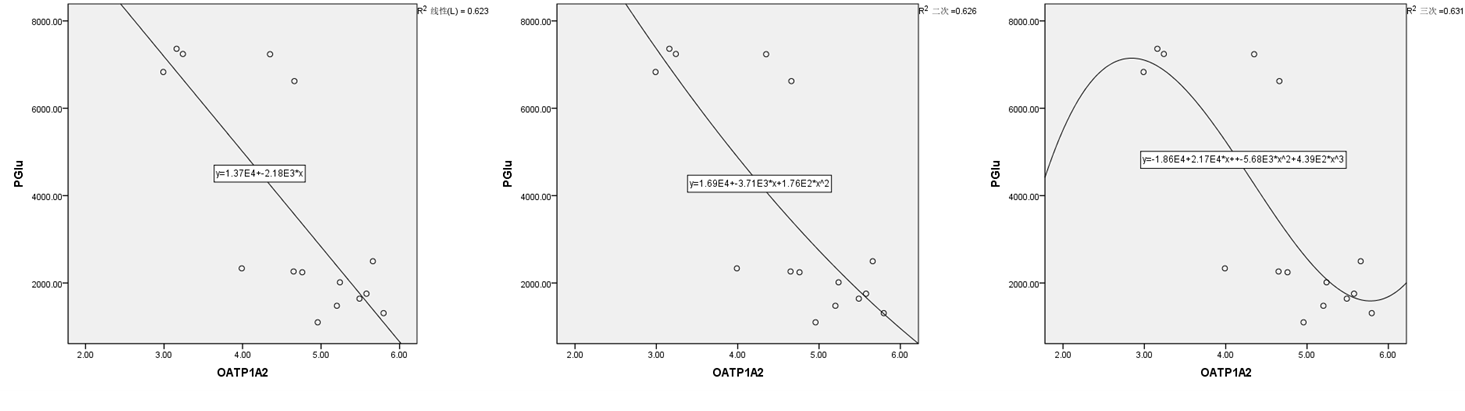
**Supplementary Figure 20** The correlation between the expression level of OATP1A2 and the AUC of P-Glu (*p*<0.01)


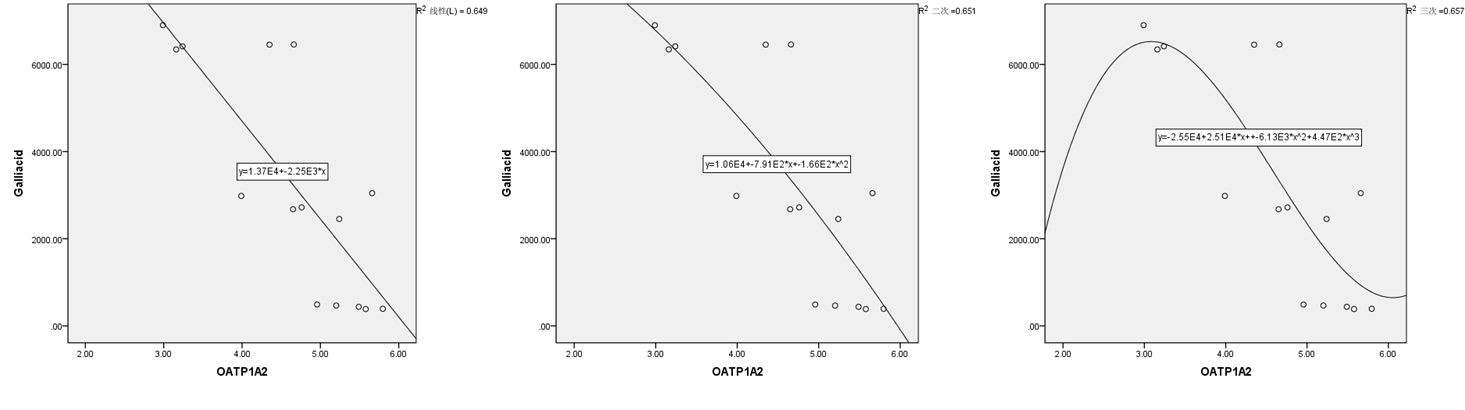
**Supplementary Figure 21** The correlation between the expression level of OATP1A2 and the AUC of gallic acid (*p*<0.01)


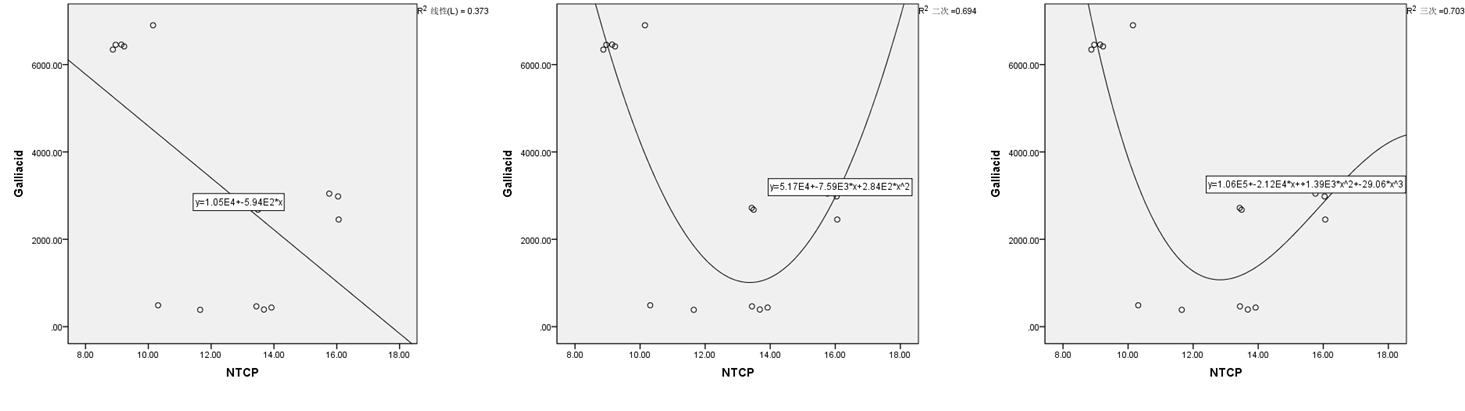
**Supplementary Figure 22** The correlation between the expression level of NTCP and the AUC of gallic acid (*p*<0.05)


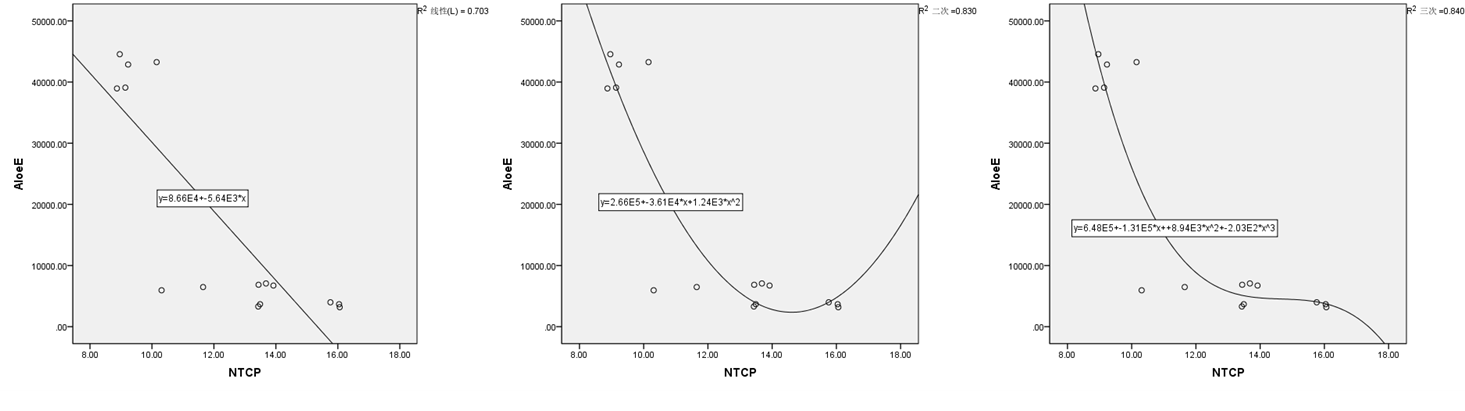
**Supplementary Figure 23** The correlation between the expression level of NTCP and the AUC of aloe-emodin (*p*<0.01)


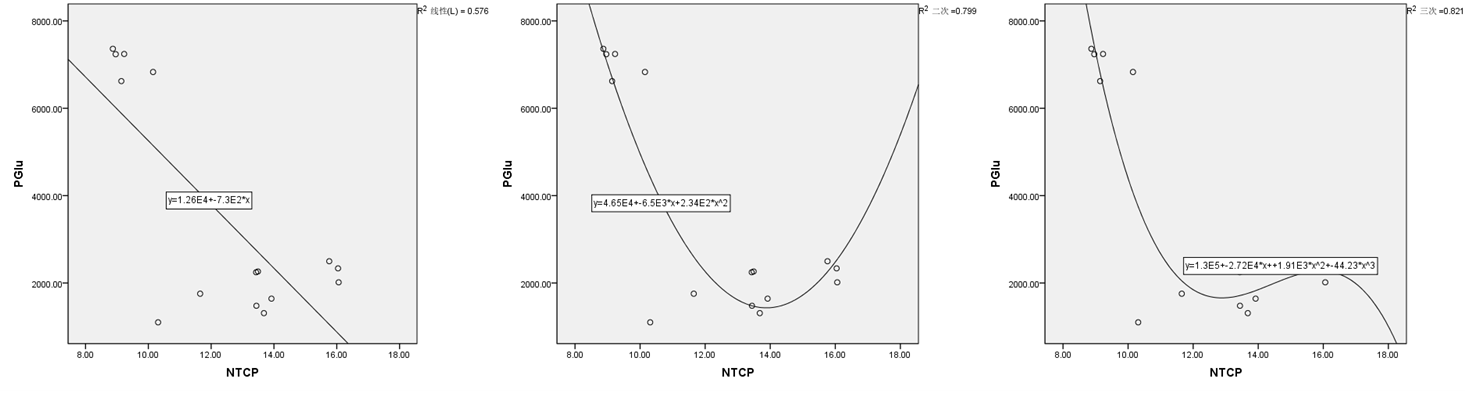


**Supplementary Figure 24** The correlation between the expression level of NTCP and the AUC of P-Glu (*p*<0.05)


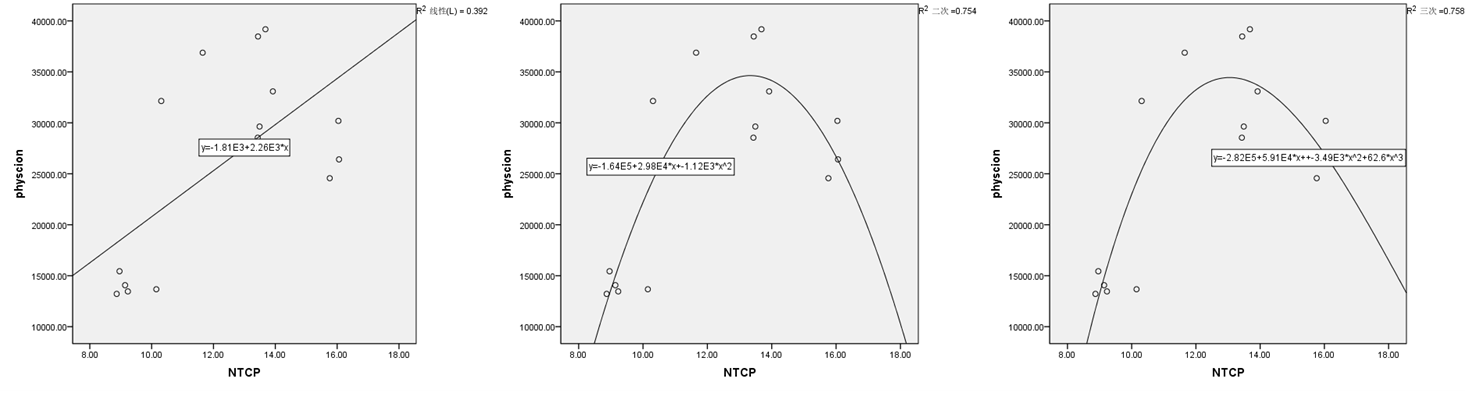


**Supplementary Figure 25** The correlation between the expression level of NTCP and the AUC of physcion (*p*<0.05)


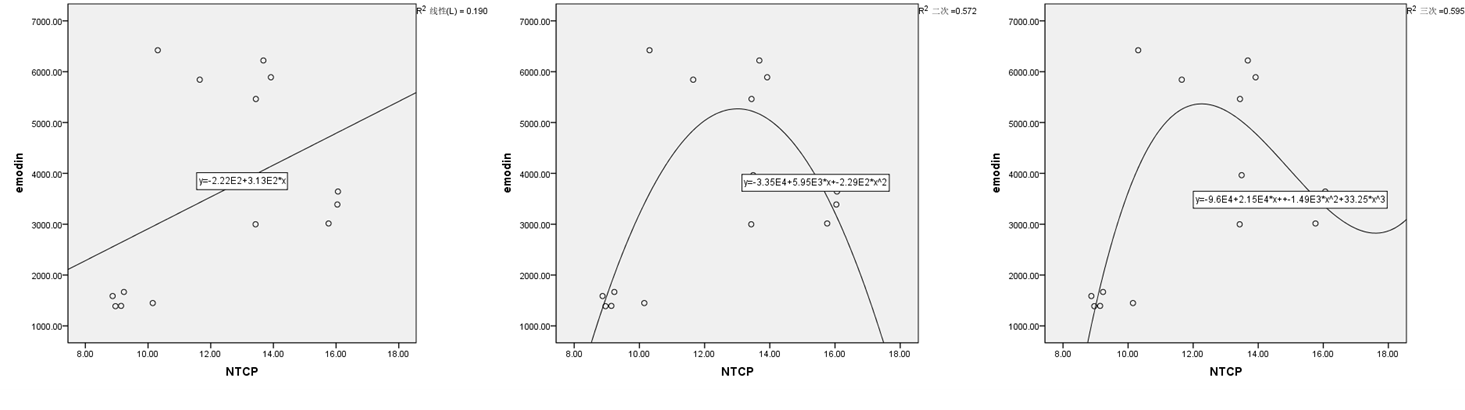
**Supplementary Figure 26** The correlation between the expression level of NTCP and the AUC of emodin (*p*<0.05)


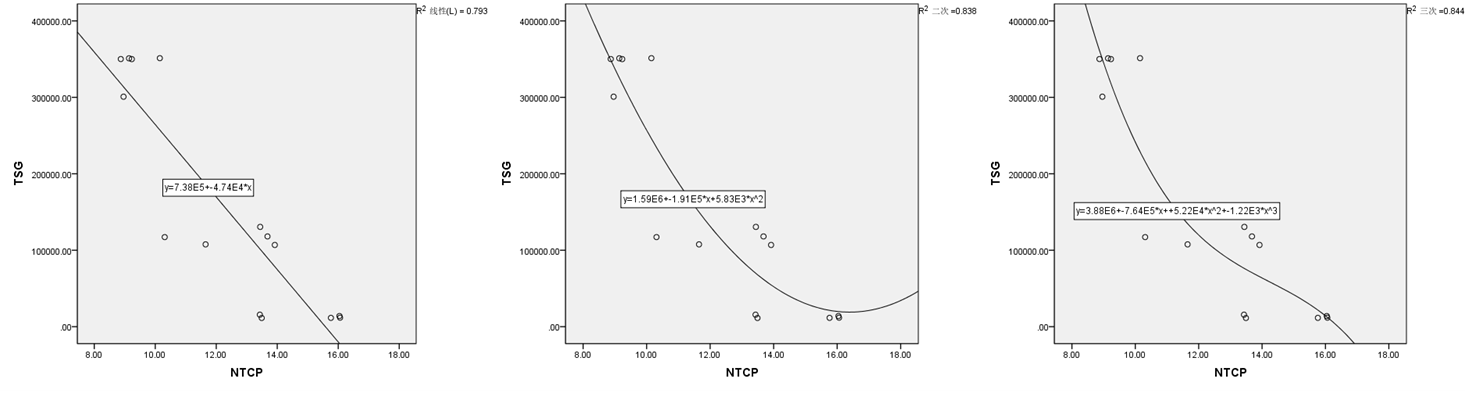


**Supplementary Figure 27** The correlation between the expression level of NTCP and the AUC of TSG (*p*<0.01)


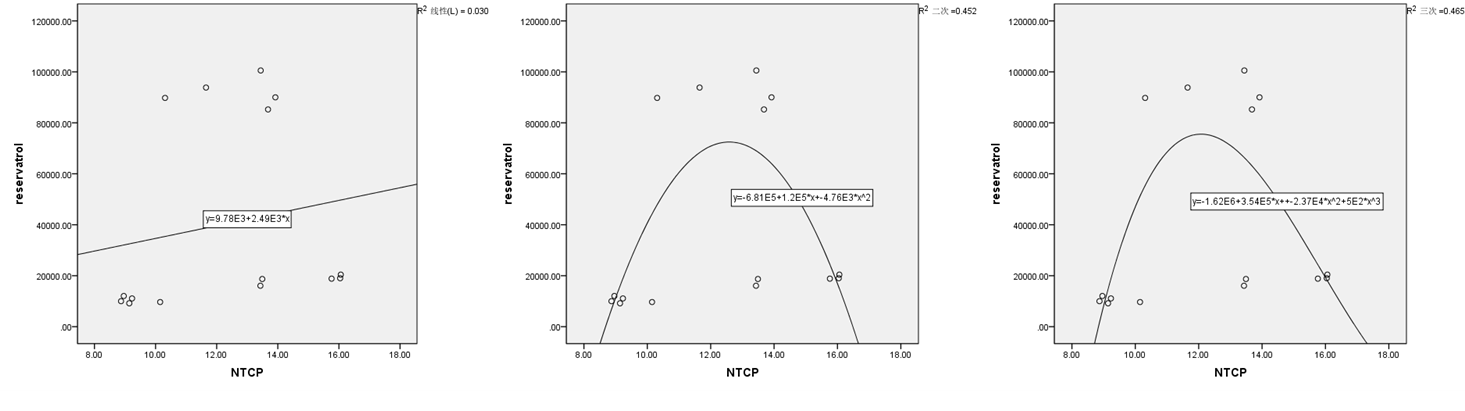


**Supplementary Figure 28** The correlation between the expression level of NTCP and the AUC of phase II metabolites of resveratrol (*p*<0.05)


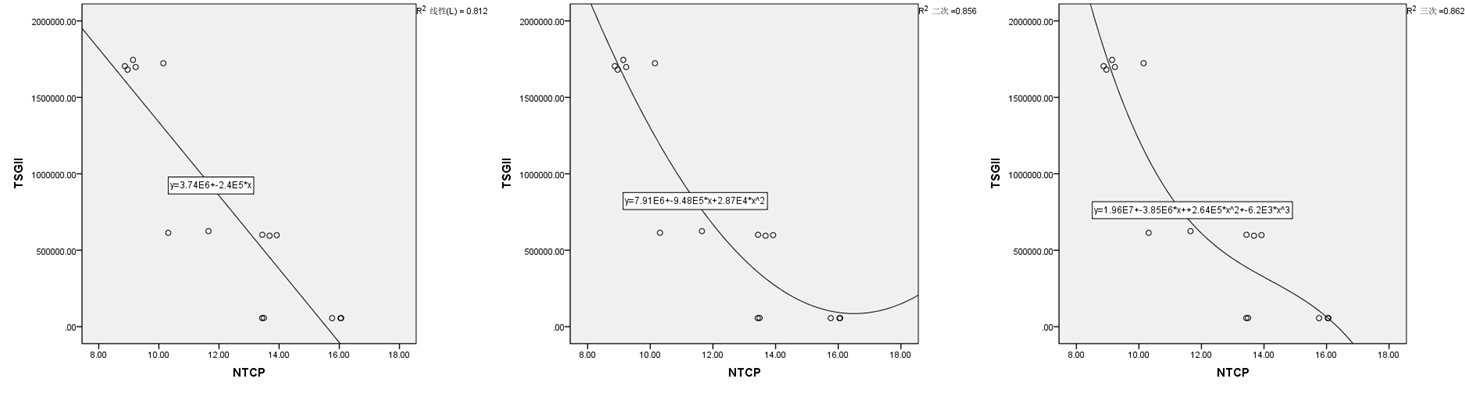


**Supplementary Figure 29** The correlation between the expression level of NTCP and the AUC of phase II metabolites of TSG (*p*<0.01)


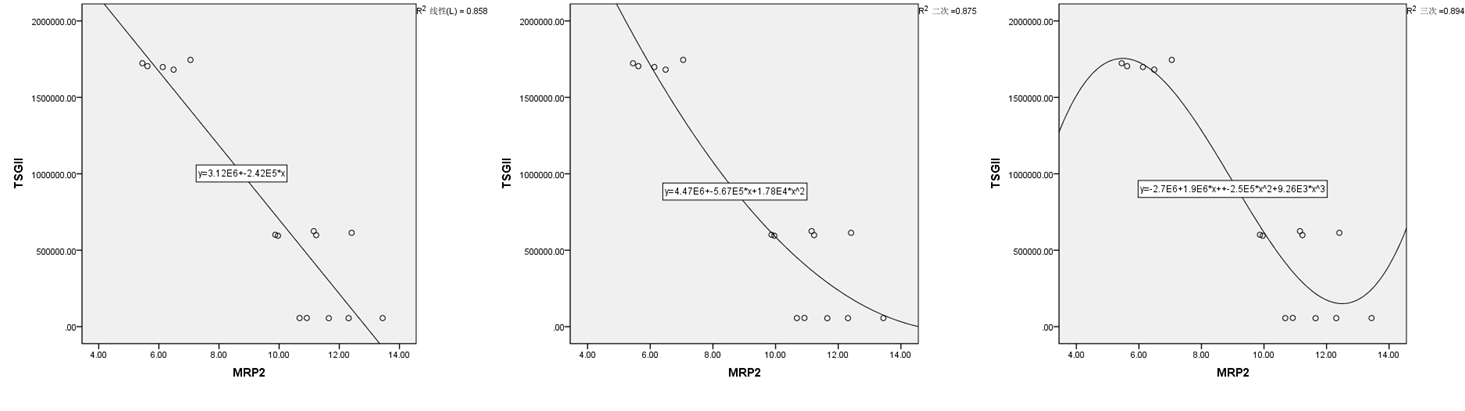


**Supplementary Figure 30** The correlation between the expression level of MRP2 and the AUC of phase II metabolites of TSG (*p*<0.01)


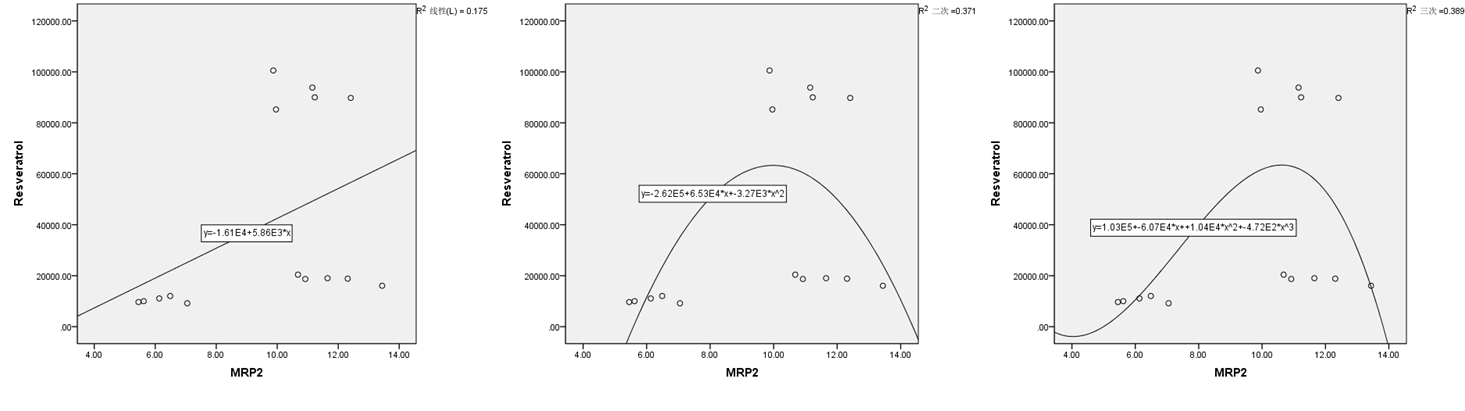


**Supplementary Figure 31** The correlation between the expression level of MRP2 and the AUC of phase II metabolites of resveratrol (*p*<0.05)


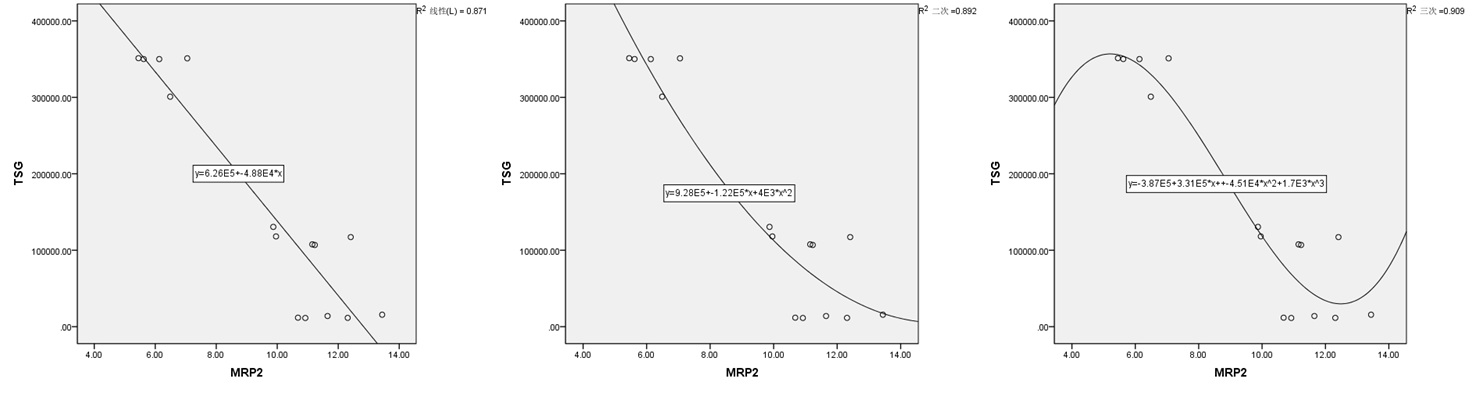


**Supplementary Figure 32** The correlation between the expression level of MRP2 and the AUC of TSG (*p*<0.01)


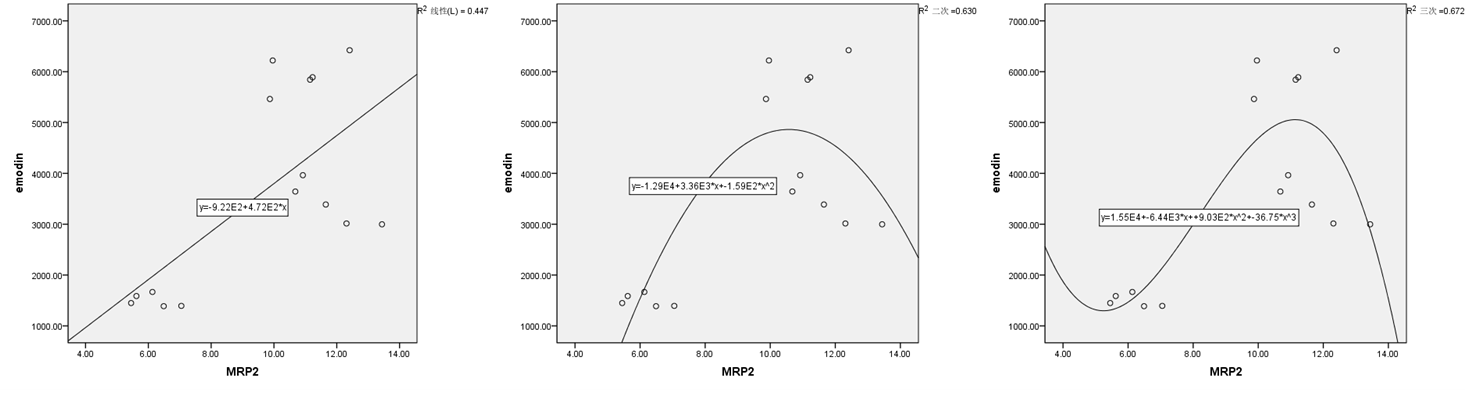
**Supplementary Figure 33** The correlation between the expression level of MRP2 and the AUC of emodin (*p*<0.05)


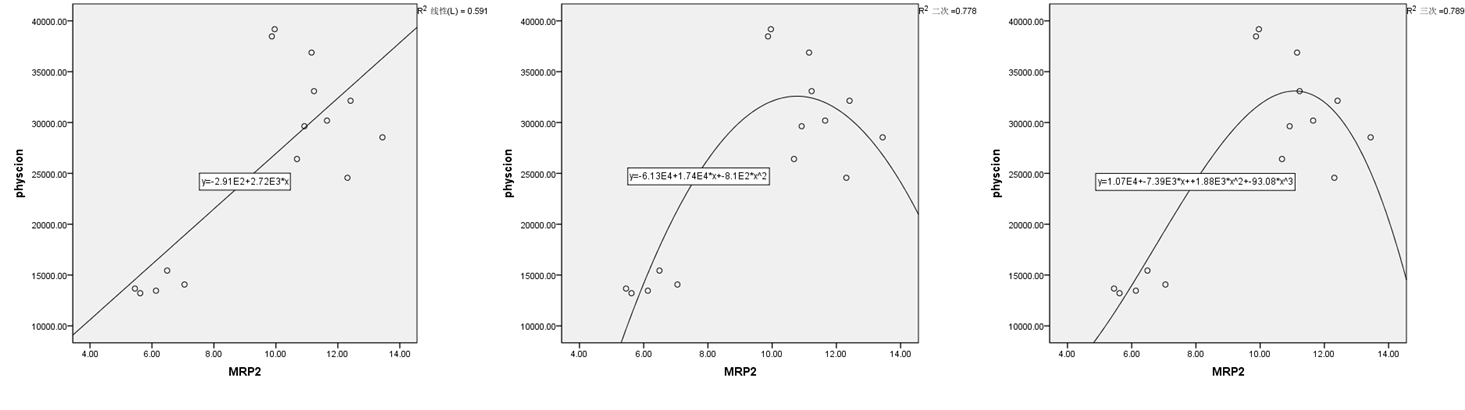


**Supplementary Figure 34** The correlation between the expression level of MRP2 and the AUC of physcion (*p*<0.05)


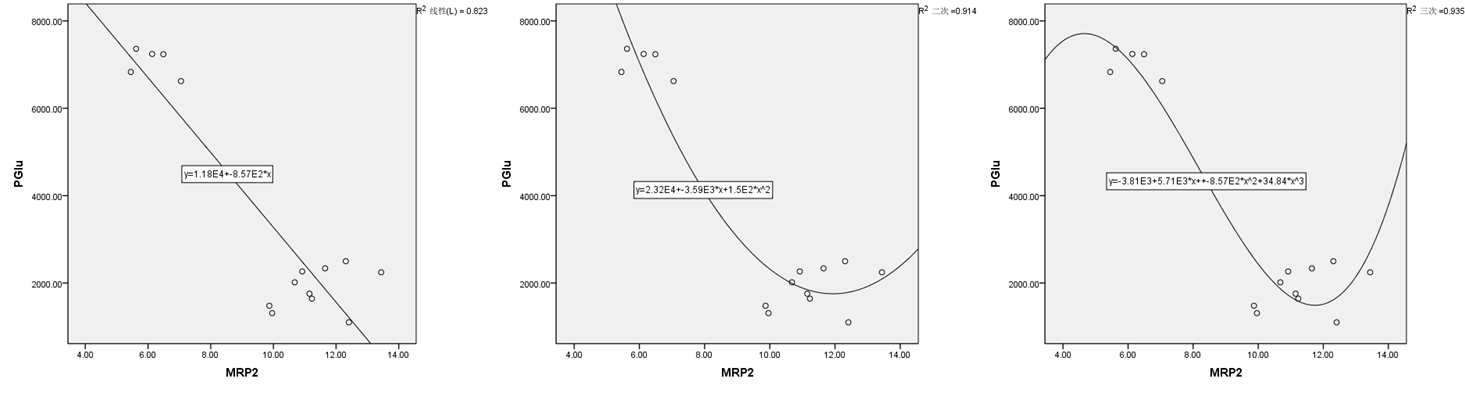
**Supplementary Figure 35** The correlation between the expression level of MRP2 and the AUC of P-Glu (*p*<0.05)


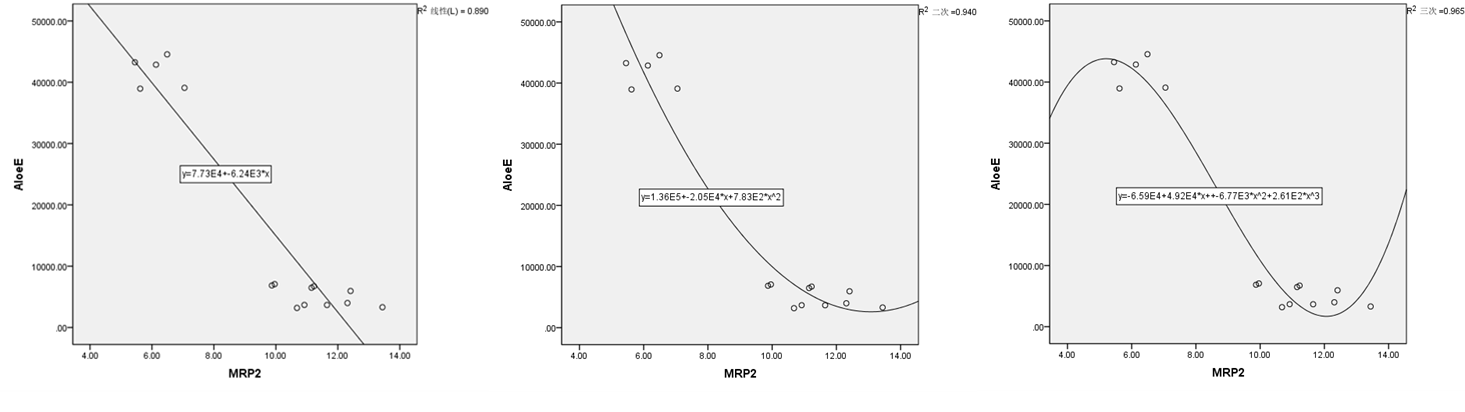
**Supplementary Figure 36** The correlation between the expression level of MRP2 and the AUC of aloe-emodin (*p*<0.01)


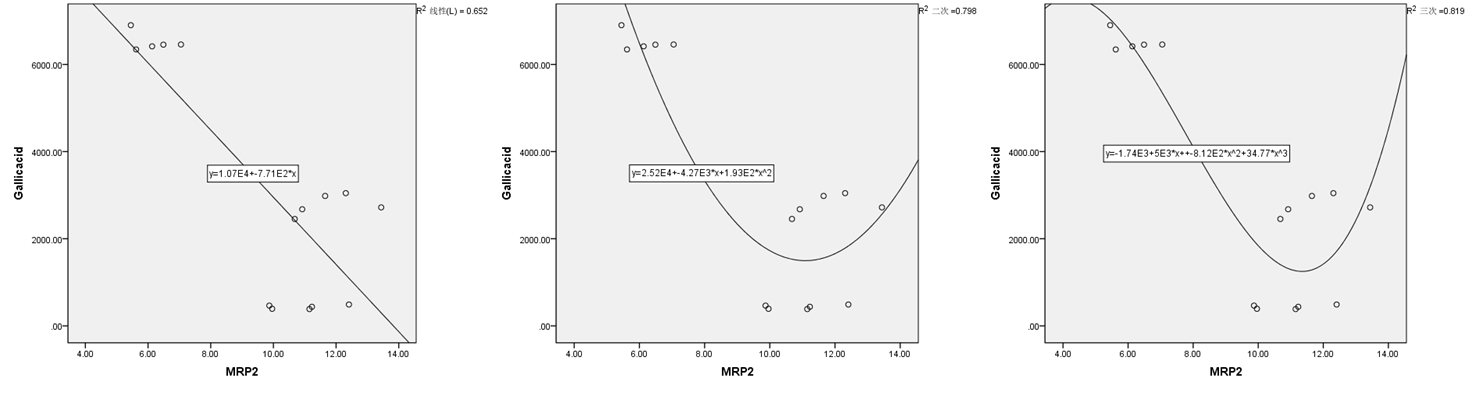


**Supplementary Figure 37** The correlation between the expression level of MRP2 and the AUC of gallic acid (*p*<0.05)


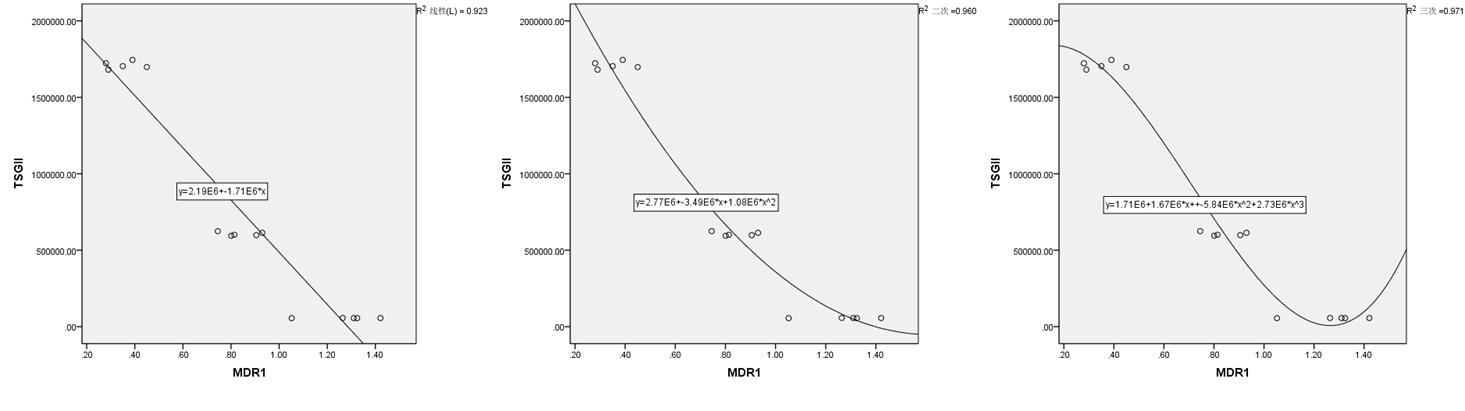


**Supplementary Figure 38** The correlation between the expression level of MDR1 and the AUC of phase II metabolites of TSG (*p*<0.01)


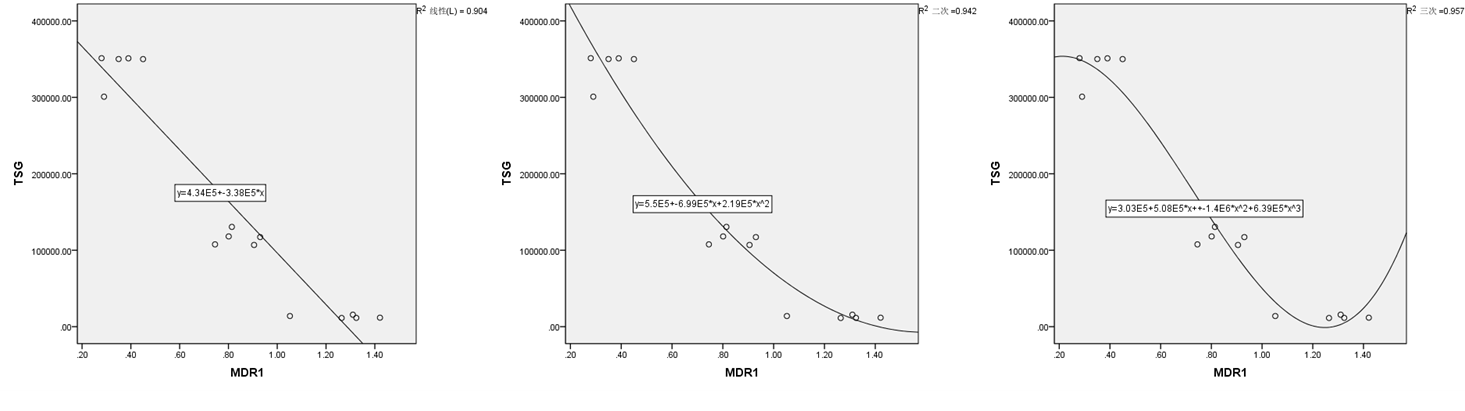


**Supplementary Figure 39** The correlation between the expression level of MDR1 and the AUC of TSG (*p*<0.01)


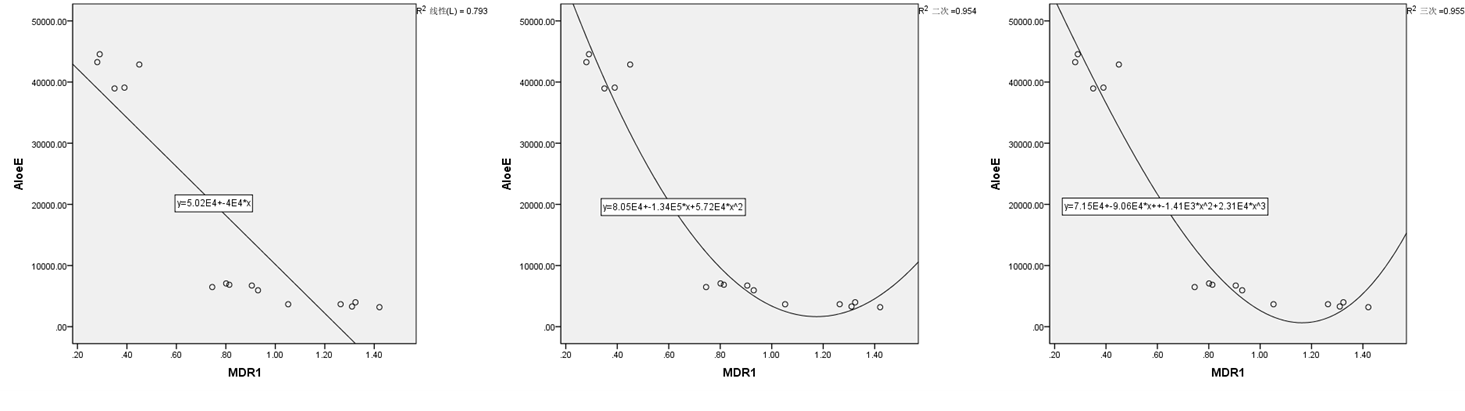


**Supplementary Figure 40** The correlation between the expression level of MDR1 and the AUC of aloe-emodin (*p*<0.01)


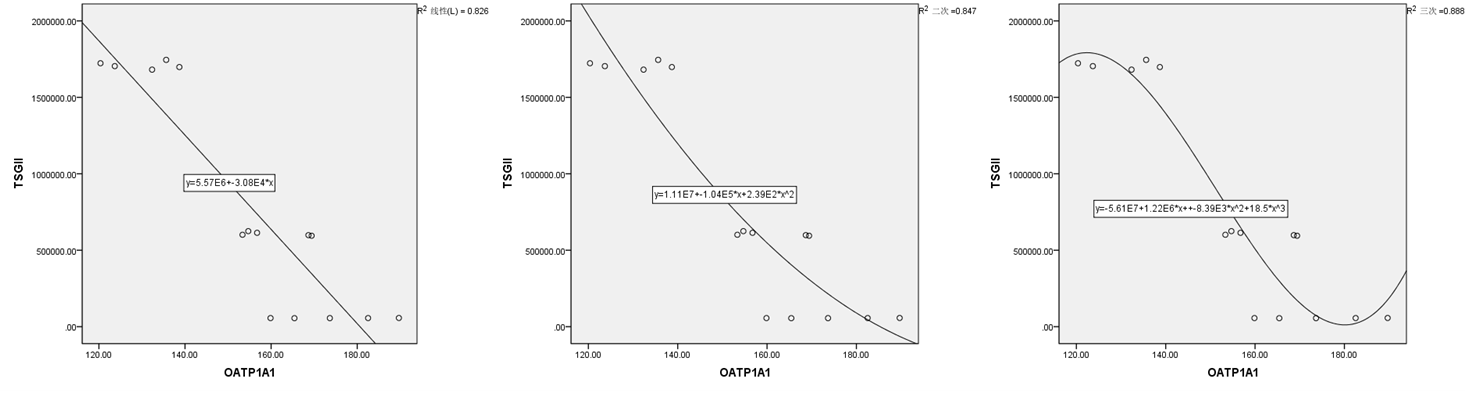
**Supplementary Figure 41** The correlation between the expression level of OATP1A1 and the AUC of phase of metabolites of TSG (*p*<0.01)


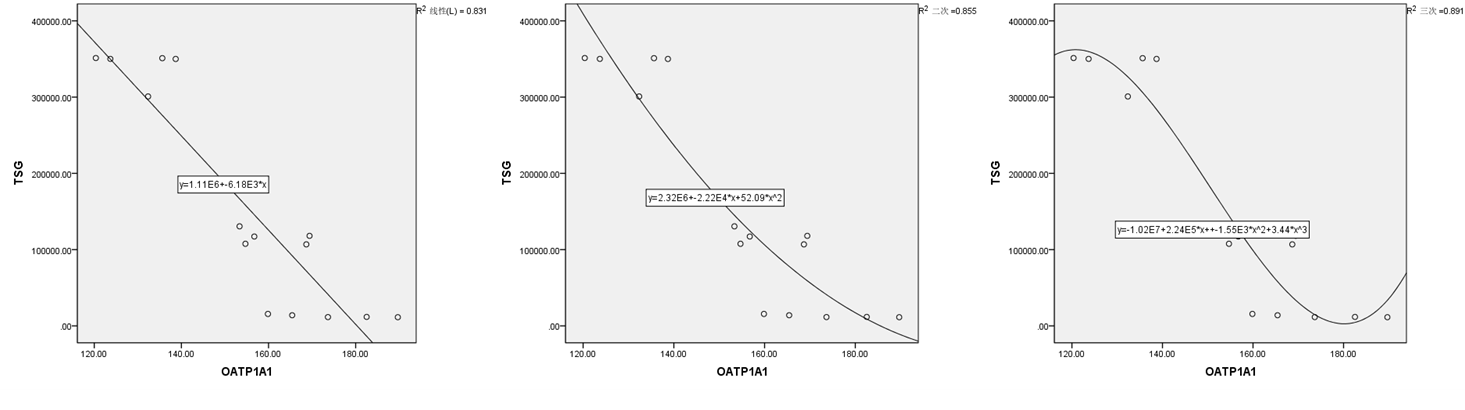
**Supplementary Figure 42** The correlation between the expression level of OATP1A1 and the AUC of TSG (*p*<0.01)


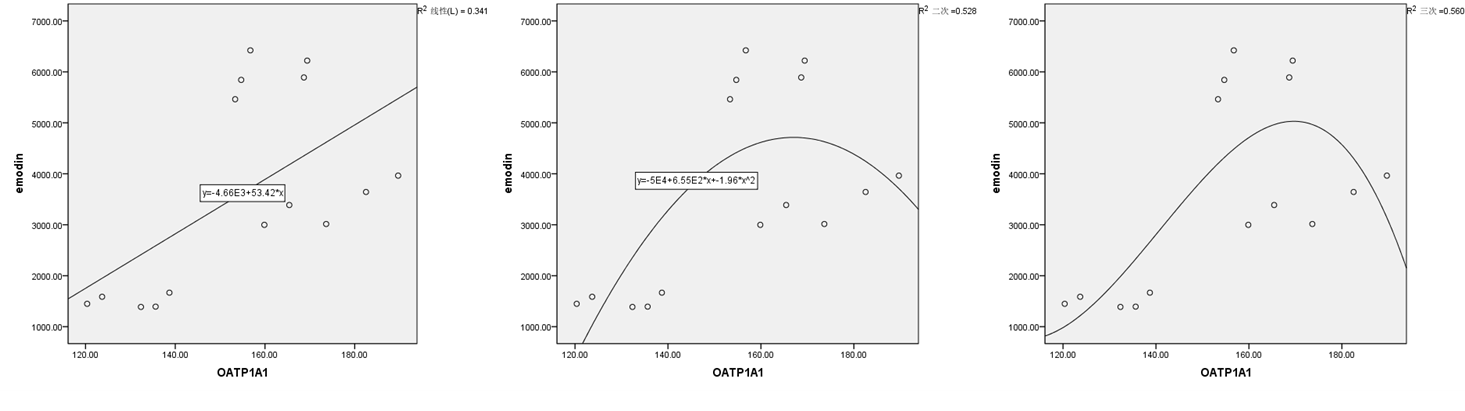


**Supplementary Figure 43** The correlation between the expression level of OATP1A1 and the AUC of emodin (*p*<0.05)


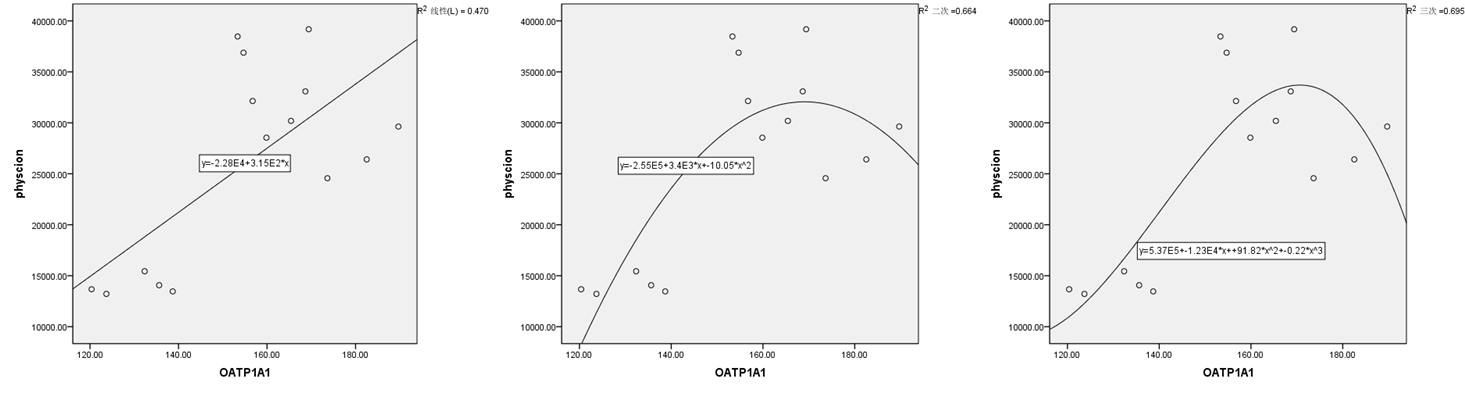


**Supplementary Figure 44** The correlation between the expression level of OATP1A1 and the AUC of physcion (*p*<0.05)


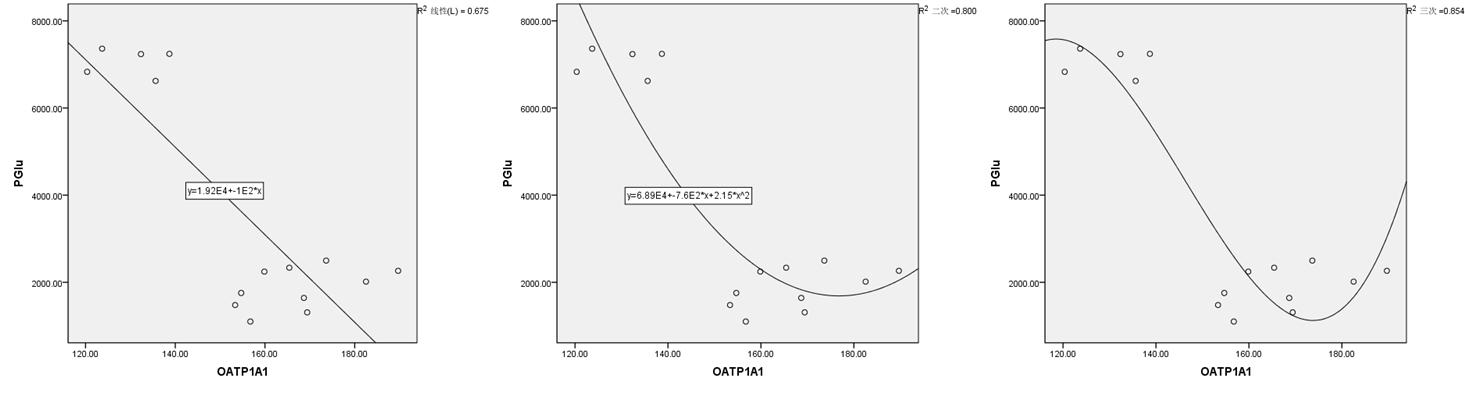


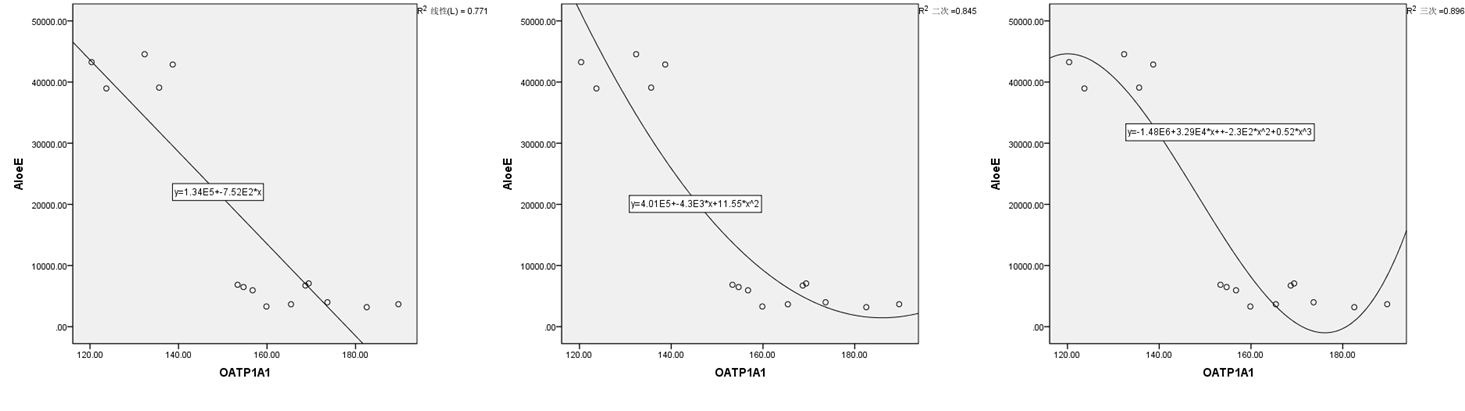


**Supplementary Figure 46** The correlation between the expression level of OATP1A1 and the AUC of aloe-emodin (*p*<0.01)


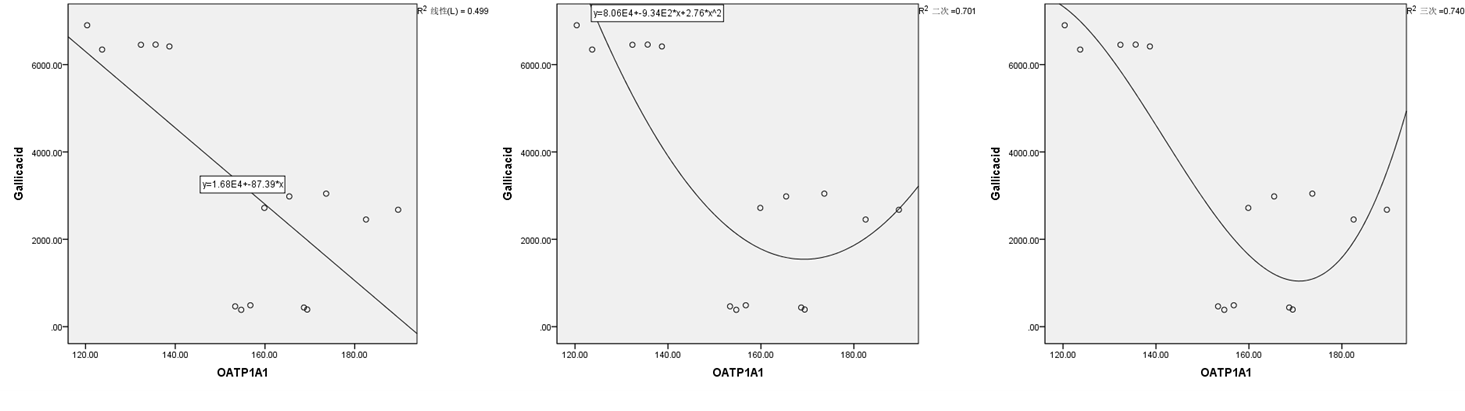
**Supplementary Figure 47** The correlation between the expression level of OATP1A1 and the AUC of gallic acid (*p*<0.05)


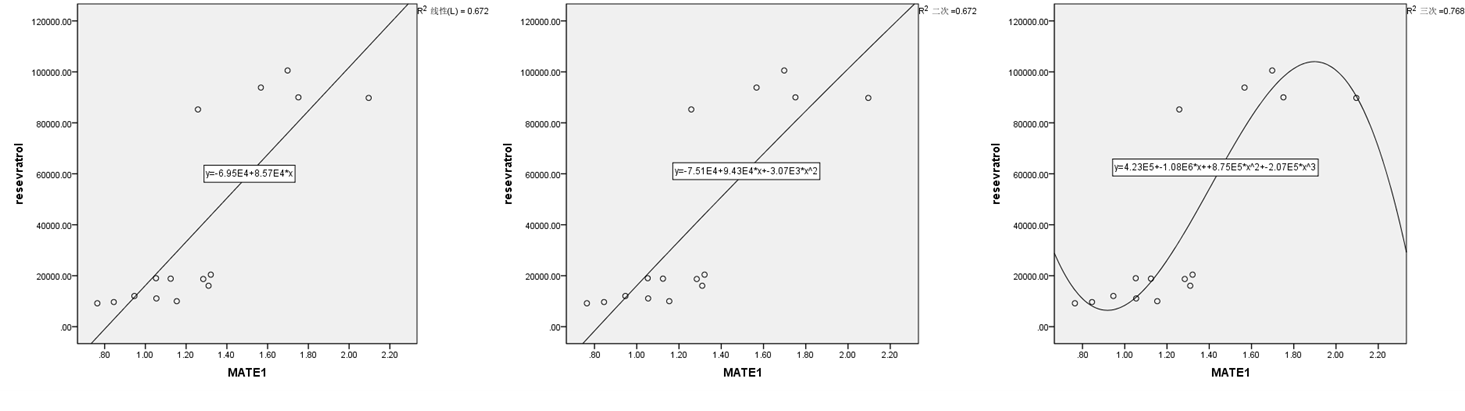
**Supplementary Figure 48** The correlation between the expression level of MATE1 and the AUC of phase II metabolites of resveratrol (*p*<0.01)


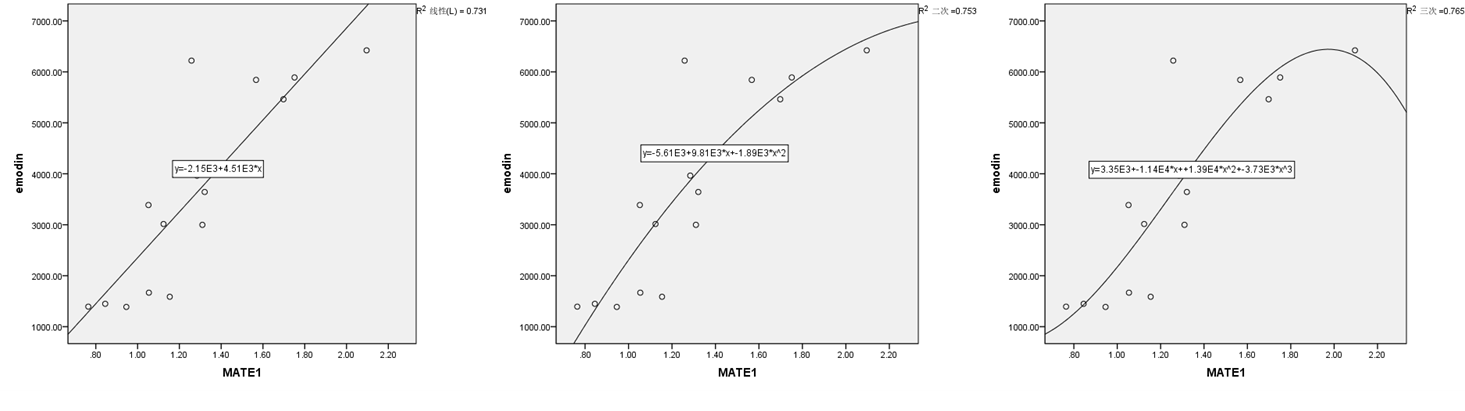
**Supplementary Figure 49** The correlation between the expression level of MATE1 and the AUC of emodin (*p*<0.01)


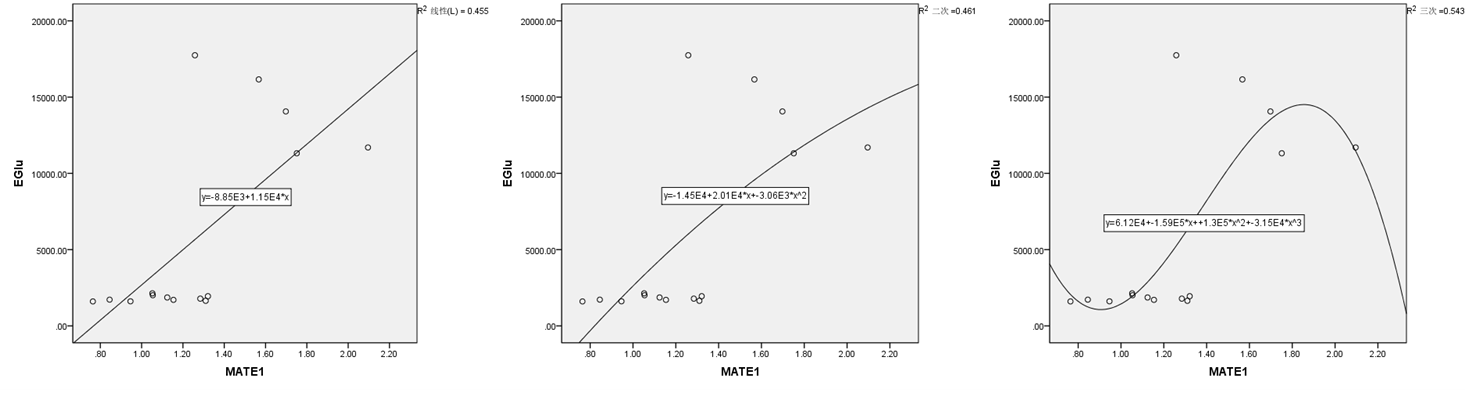


**Supplementary Figure 50** The correlation between the expression level of MATE1 and the AUC of E-Glu (*p*<0.05)


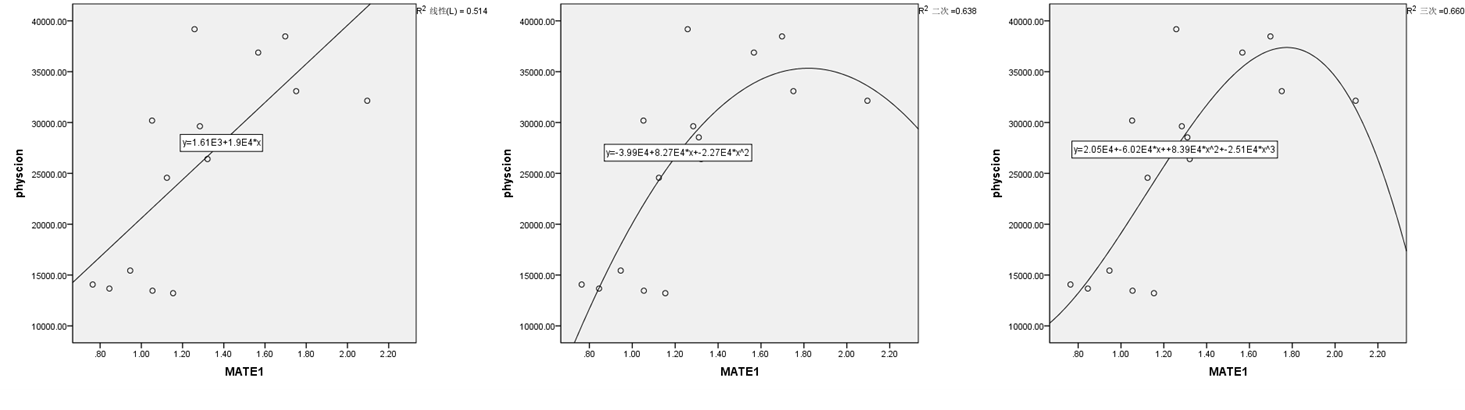
**Supplementary Figure 51** The correlation between the expression level of MATE1 and the AUC of physcion (*p*<0.01)


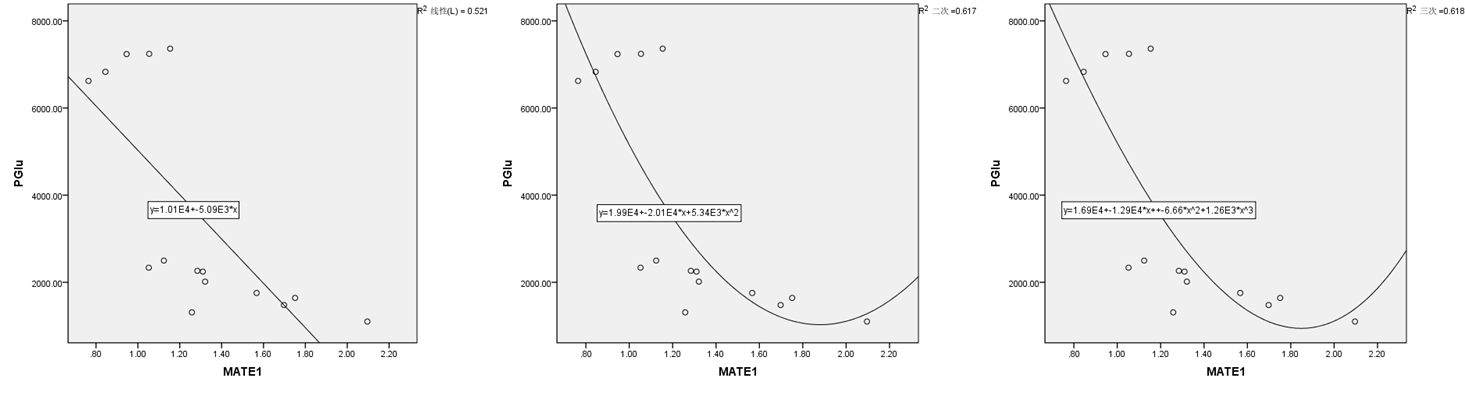
**Supplementary Figure 52** The correlation between the expression level of MATE1 and the AUC of P-Glu (*p*<0.01)


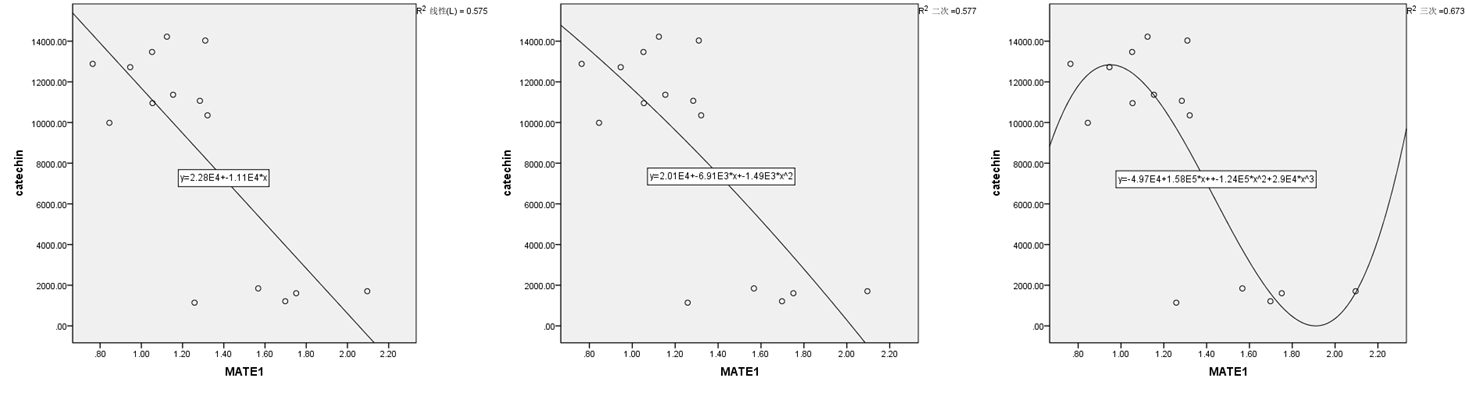
**Supplementary Figure 53** The correlation between the expression level of MATE1 and the AUC of catechin (*p*<0.05)


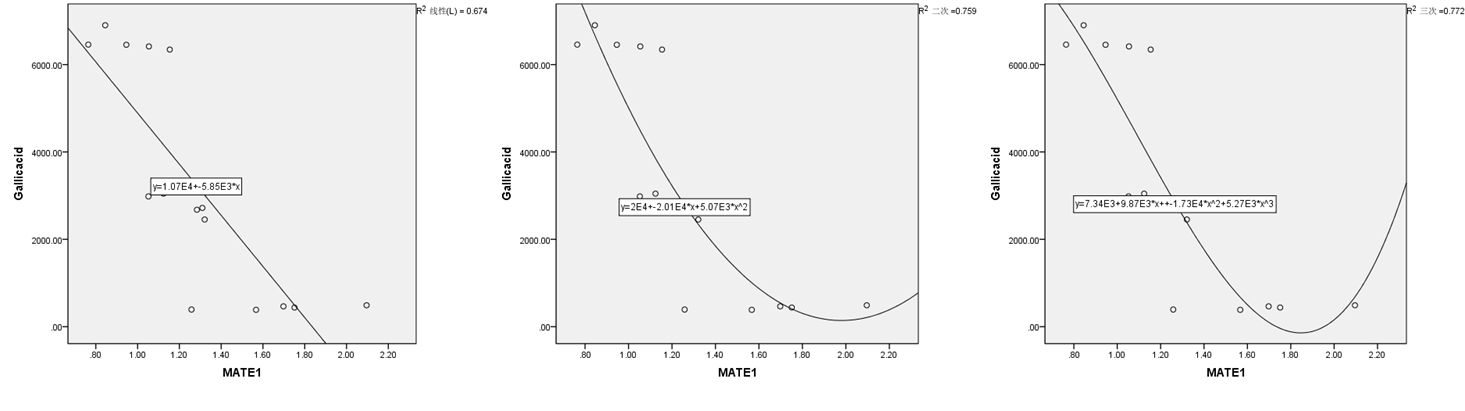


**Supplementary Figure 54** The correlation between the expression level of MATE1 and the AUC of gallic acid (*p*<0.01)
